# Anatomical limitations in adventitious root formation revealed by magnetic resonance imaging, infrared spectroscopy, and histology of rose genotypes with contrasting rooting phenotypes

**DOI:** 10.1093/jxb/erae158

**Published:** 2024-04-12

**Authors:** David Wamhoff, André Gündel, Steffen Wagner, Stefan Ortleb, Ljudmilla Borisjuk, Traud Winkelmann

**Affiliations:** Institute of Horticultural Production Systems, Section Woody Plant and Propagation Physiology, Leibniz Universität Hannover, Hannover, Germany; Leibniz-Institute of Plant Genetics and Crop Plant Research (IPK), Corrensstrasse 3, 06466 Seeland-Gatersleben, Germany; Stockholm University, Department of Ecology, Environment and Plant Sciences, Svante Arrhenius Väg 21 A Frescati Backe Stockholm SE-106 91, Sweden; Leibniz-Institute of Plant Genetics and Crop Plant Research (IPK), Corrensstrasse 3, 06466 Seeland-Gatersleben, Germany; Leibniz-Institute of Plant Genetics and Crop Plant Research (IPK), Corrensstrasse 3, 06466 Seeland-Gatersleben, Germany; Leibniz-Institute of Plant Genetics and Crop Plant Research (IPK), Corrensstrasse 3, 06466 Seeland-Gatersleben, Germany; Institute of Horticultural Production Systems, Section Woody Plant and Propagation Physiology, Leibniz Universität Hannover, Hannover, Germany; Ikerbasque, Spain

**Keywords:** Cell wall composition, 3D imaging, nuclear magnetic resonance, *Rosa hybrida*, stem cutting

## Abstract

Adventitious root (AR) formation is one of the most important developmental processes in vegetative propagation. Although genotypic differences in rose rooting ability are well known, the causal factors are not well understood. The rooting of two contrasting genotypes, ‘Herzogin Friederike’ and ‘Mariatheresia’, was compared following a multiscale approach. Using magnetic resonance imaging, we non-invasively monitored the inner structure of stem cuttings during initiation and progression of AR formation for the first time. Spatially resolved Fourier-transform infrared spectroscopy characterized the chemical composition of the tissues involved in AR formation. The results were validated through light microscopy and complemented by immunolabelling. The outcome demonstrated similarity of both genotypes in root primordia formation, which did not result in root protrusion through the shoot cortex in the difficult-to-root genotype ‘Mariatheresia’. The biochemical composition of the contrasting genotypes highlighted main differences in cell wall-associated components. Further spectroscopic analysis of 15 contrasting rose genotypes confirmed the biochemical differences between easy- and difficult-to-root groups. Collectively, our data indicate that it is not the lack of root primordia limiting AR formation in these rose genotypes, but the firmness of the outer stem tissue and/or cell wall modifications that pose a mechanical barrier and prevent root extension and protrusion.

## Teaser

Combining histology, magnetic resonance imaging, and infrared spectroscopy unravelled differences in cell wall composition of stem parenchyma as causes for limitations of adventitious root emergence and successful rooting in rose.

## Introduction

Wound-induced adventitious root (AR) formation is a highly complex physiological process that is a pre-requisite to successful autovegetative propagation via cuttings, for numerous important species in the horticulture sector (Steffens and Rasmussen, 2016; Druege *et al.*, 2019). Rose is one of the most important ornamental crops in different market segments, such as garden plants, pot plants, as well as cut flowers (International Association of Horticultural Producers, 2021). While pot roses are already frequently propagated autovegetatively via cuttings (Miguel Costa *et al.*, 2017), this propagation method is limited for a large number of genotypes, in particular in the cut and garden rose segments, due to strong genetic differences in ability of AR formation (Nguyen *et al.*, 2020;  Wamhoff *et al.*, 2023). Therefore, costly budding and grafting techniques are still common in these two segments (Miguel Costa *et al.*, 2017).

The process of AR formation includes three major phases, i.e. induction, initiation, and expression, that follow the facultative phase of dedifferentiation. Dedifferentiation takes place in case the AR founder cells have to access the capability of reacting to an induction signal prior to entering the AR formation process itself (Bellini *et al.*, 2014; Druege *et al.*, 2019). In the induction phase, even microscopically undetectable changes can occur at the cellular level (De Klerk *et al.*, 1999; Druege *et al.*, 2019). Changes in plant hormone homeostasis and signalling are characteristic for this early phase of AR formation (Druege *et al.*, 2016). In contrast, recognizable cell division processes take place in the initiation phase, leading to the formation of microscopically visible meristematic cell clusters, which in the course of time develop into the so-called root primordia (RPs) with their characteristic dome-shaped appearance (De Klerk *et al.*, 1999; Druege *et al.*, 2019). ARs can originate from different cell types, which are usually associated with the vascular system of the stem, such as the cambium or the phloem parenchyma (da Costa *et al.*, 2013). The final phase of expression is entered as soon as the dome-shaped RPs develop to complete vascularized root bodies (Druege *et al.*, 2019). This phase is characterized by further cell divisions and cell elongation and the emergence of ARs (Bellini *et al.*, 2014; Lin and Sauter, 2019). The duration of the different phases of the AR formation process depends on the species or even genotype, but also on the vegetative propagation system and plant material used, and on other exogenous factors such as temperature (De Klerk *et al.*, 1999; Ahkami *et al.*, 2009; Almeida *et al.*, 2017; Miguel Costa *et al.*, 2017). Among this multitude of influencing factors, the genetic ability to form ARs is described as the strongest determinant for successful AR formation (Bellini *et al.*, 2014; Druege *et al.*, 2019).

This genetically controlled rooting ability is a result of multiple processes, such as wound responses, pronounced changes in plant hormone homeostasis, and disruption of water and nutrient supply from the mother plant directly after cut off (Ahkami *et al.*, 2009; Li *et al.*, 2009; De Klerk *et al.*, 2011; Morales-Orellana *et al.*, 2022). Genetic studies also revealed the involvement of genes that are known to play a role in processes such as stress responses, cell division, or cell wall processing in different phases of AR formation (Druege *et al.*, 2019; Li, 2021). However, the role of the internal environment, such as structural and biochemical features of stem tissues instantly involved in AR formation, as well as anatomical rearrangements occurring in the surrounding of the developing ARs, are less considered (Hartmann *et al.*, 2014; Díaz-Sala, 2020).

Thus, anatomical investigations along with spatially resolved characterization of tissue chemistry, could make important contributions to identifying factors influencing AR formation. Technologies such as high-mass-resolution MALDI mass spectrometry (Sarabia *et al.*, 2018), Fourier-transform infrared (FTIR) spectroscopy (Gündel *et al.*, 2021), as well as classical immunochemistry using confocal laser scanning microscopy (Pegg *et al.*, 2020) localize and quantify specific molecules or functional groups with high spatial resolution in plant tissues. An important aspect is the integration of available classical and powerful modern approaches for a better understanding of the processes underlying the visual phenotype.

The objective of this study was the identification of factors that limit the formation of AR in roses, both structurally and biochemically. To achieve this, we employed non-invasive magnetic resonance imaging (MRI) and high-resolution FTIR imaging techniques and examined AR formation in stem cuttings of two divergent rose genotypes: ‘Mariatheresia’ (MT, which is difficult to root) and ‘Herzogin Friederike’ (HF, which is easy to root). Our findings reveal new and distinctive features associated with the rooting ability of these two genotypes, which may have implications for other rose varieties tested under similar conditions. Our results open up new perspectives regarding the role and the fate of the cell layers surrounding RPs, which should be considered to improve this key step in vegetative propagation of many plant species, especially woody plants.

## Materials and methods

### Plant material and *in vitro* culture conditions

A total of 15 rose genotypes, including HF and MT (Supplementary Table S1), were used in this study. The genotypes were selected based on their ability to form ARs, which was recorded previously (Nguyen *et al.*, 2020). An overview of the plant material that was used for the different experimental approaches to study differences in AR formation is provided in Supplementary Fig. S1. Shoots were cultured *in vitro* for proliferation in full-strength Murashige and Skoog (MS) salts and vitamins (Murashige and Skoog, 1962) but with FeEDTA replaced by 231 µM FeEDDHA, 30 g l^–1^ sucrose, 2.21 μM 6-benzylaminopurine, 0.29 μM gibberellic acid 3, and 8 g l^–1^ Plant agar (Duchefa, Harlem, Netherlands). Plant material was cultivated under fluorescent light tubes at a photon flux density (PAR) of 40 µmol m^–2^ s^–1^ at 24 °C (± 2 °C) and 16 h photoperiod in 250 ml polypropylene vessels (Plastikbecher.de GmbH, Giengen, Germany). The shoot cultures were transferred onto fresh medium every 4–5 weeks.

To study AR formation characteristics in different experiments, apical shoots of 1–1.5 cm length were excised at the end of a 4 weeks cultivation cycle for proliferation. Apical shoots were transferred into 250 ml polypropylene vessels (Plastikbecher.de GmbH, Giengen, Germany), containing rooting medium consisting of half strength MS macro- and microelements and full-strength MS vitamins (Murashige and Skoog, 1962), 20 g l^–1^ sucrose, and 7.5 g l^–1^ Plant agar (Duchefa, Harlem, Netherlands). Depending on the experiment, 0.98 µM IBA (indole-3-butyric acid, +IBA) was added, or the rooting medium stayed IBA-free (–IBA). The pH of all culture media was adjusted to 5.8 and media were autoclaved at 121 °C and 2000 hPa for 20 min.

### Morphological analysis of AR formation

Five vessels with six shoots each for genotypes HF and MT were cultured under the described conditions for 21 d on +IBA or –IBA medium. The experiment was repeated twice. Rooting (recorded as yes/no) was evaluated weekly after 7, 14, and 21 d, with AR formation being defined in this and following experiments as a clearly distinguishable root structure that had emerged through the stem epidermis. Additionally, root number and root length were analysed within a destructive final evaluation after 21 d. Roots were considered as such and analysed if they had a length of at least 2 mm. To determine the root length, roots were excised and scanned (STD4800 scanner, calibrated for WinRHIZO image analyses, Regent Instruments Inc., Quebec, Canada) using WinRHIZO (Basic version 2019a, Regent Instruments Inc., Quebec, Canada). Lengths of single roots per rooted shoots were measured using ImageJ (version 2.9.0/1.53t, Rueden *et al.*, 2017) by freehand line measurement.

### Histological analysis of root primordia development

In total, 15 shoots each were cultivated on +IBA and –IBA medium, divided on three 250 ml vessels, each for genotypes HF and MT. After 7 d of cultivation, shoot bases were cut, rinsed in deionized water, and fixed in alcohol-formalin-acetic acid (AFA), prior to an EtOH dehydration series and paraffin embedding into ROTI®Plast (Carl Roth GmbH + Co. KG, Karlsruhe, Germany). The AFA fixative was replaced with 70% ethanol, and shoot bases were stored at 4 °C until use. For histological analysis, the shoot bases were rinsed in deionized water and transferred to fresh 70% EtOH overnight, followed by an increasing series of EtOH solutions [70%, 80%, 90%, and 96% EtOH (v/v) for 10, 15, 30, and 30 min, respectively] and two incubations in 100% isopropanol for 40 min each. Infiltration started by incubating the samples twice in xylene substitute ROTI®Clear (Carl Roth GmbH + Co. KG, Karlsruhe, Germany) for 40 min each, followed by infiltration in xylene substitute: paraffin (ROTI®Plast, Carl Roth GmbH + Co. KG, Karlsruhe, Germany) mixture (1:1, v/v) at 62 °C. Infiltration ended with two steps of 40 min incubation in ROTI®Plast at 62 °C. All dehydration and infiltration steps were conducted under vacuum of 11 kPa. Shoot bases were embedded in polyvinyl chloride embedding moulds (7 × 7 × 5 mm, PLANO GmbH, Wetzlar, Germany) in ROTI®Plast. A polypropylene mesh with a mesh size of 1 mm or 2 mm (dependent on explant size; Franz Eckert GmbH, Waldkirch, Germany) was used to stabilize the stem base in the mould in an upright position during the cooling process on ice. Cross-sections were cut 7–10 µm thick with a rotation microtome (Hydrax M 55, Zeiss, Oberkochen, Germany) and transferred via a water bath onto glass microscopic slides. Sections were dewaxed, rehydrated, and serially stained with rhodamine B, acriflavine, and astra blue (RAA): The sections were dewaxed in xylene substitute (twice, 10 min each) and rehydrated in a graded EtOH series (96%, 80%, 70%, 60% EtOH, 10 min each) followed by two steps in deionized water for 5 min each. Rehydrated cross sections were serially RAA-stained with rhodamine B solution (1%, dissolved in 50% EtOH, Kremer Pigmente GmbH & Co. KG, Aichstetten, Germany) for 10 min, aqueous acriflavine solution (1% pre-mixed, MOPRHISTO GmbH, Offenbach a.M., Germany) for 10 s, followed by aqueous astra blue (1%, pre-mixed, MOPRHISTO GmbH, Offenbach a.M., Germany) for 2 min [staining procedure modified from Wacker (2006)]. The cross sections were rinsed in deionized water two to three times after each staining step. Finally, the sections were incubated in 100% isopropanol for 1 min. Microscopy was performed using a VHX-7000 digital microscope (KEYENCE Deutschland GmbH, Neu-Isenburg, Germany). Cell walls are stained blue by astra blue, modified cell walls (e.g. lignified, suberized, cutinized) are stained reddish to pink mainly by rhodamine B, DNA/RNA rich regions are stained yellow by acriflavin (Wacker 2006). The number of RPs per stem base was counted in one representative, complete cross-section per shoot base (*n*=15). For individual RPs, the dimensions of width, length and area were measured. For an indication of cell dimensions, the number of crossed cell walls was counted by laying a radial 150 µm line into the cross-section, once directly in front of an RP and once in the shoot cortex in a region without any RP.

### Data analyses of rooting characteristics

Statistical analyses of data for all AR formation parameters were performed using *R* software (v4.2.1, R Core Team, 2021). Rooting data (recorded as yes/no) after 7, 14, and 21 d were used to calculate rooting percentages per vessel. Data were ordered as quantile normalized and transformed using *R* package *bestNormalize* (v1.8.3, Peterson and Cavanaugh, 2020). Transformed rooting percentages were analysed within a linear mixed model (LMM) with fixed effects repetition (rep1, rep2), genotype (HF, MT), IBA variant (+IBA, –IBA), and time after excision (days), as well as their interactions and a random effect for the vessel id to consider repeated measurements from the same vessel by using the *R* package *nlme* (v3.1-157, Pinheiro *et al.*, 2022). Root number per rooted shoot was analysed within a generalized linear model (GLM) under assumption of Poisson distribution, while the mean root length per rooted shoot was analysed in a linear model (LM) as ordered quantile normalized data, both with repetition, genotype, IBA variant and their interactions as fixed effects. The number of RPs per shoot base was analysed in a GLM with factors genotype, IBA variant, and vessel as fixed effects under assumption of quasi-Poisson distribution. Fixed factor effects in GLMs were submitted to deviance analyses and in LM and LMM to analysis of variance (ANOVA), both followed by pairwise comparisons (Tukey’s test, *P*<0.05) by using the *R* package *emmeans* (v1.8.4-1, Lenth, 2022) in case of significant effects for the fixed factors. RP dimension measurements were analysed in a LMM with fixed effect genotype and shoot base identity as random effect to consider the common origin of the RP from the same shoot base.

### Magnetic resonance imaging

Avance III HD 400 MHz NMR-spectrometer (Bruker Biospin, Rheinstetten, Germany) was used for visualization of AR formation in shoots of two genotypes HF and MT. The shoots of both genotypes were grown in parallel *in vitro* for a period of 10 d on +IBA rooting medium. The imaging of each sample was carried out in 5 mm NMR tubes at 0, 3, 5, 7, and 10 d with a resolution of 30 µm (FOV 7 × 4.4 × 4.4 mm, RT 500 ms, echo time to 7.1 ms). Data analysis was performed using Matlab (vR2019b, The MathWorks Inc., Natick, MA, USA) and AMIRA software (Amira3D 2022.1, ThermoFisher Scientific, Inc., Schwerte, Germany). After the MRI scans, the shoots were fixed in AFA for histological analysis, as described above.

### Fourier-transform infrared spectroscopy

FTIR spectroscopic analysis of pulverized samples of shoot bases was performed using the Bruker Invenio S instrument (Bruker Optics, Ettlingen, Germany). The 15 genotypes under investigation represented two groups by their ability to form ARs (easy-to-root, five genotypes; difficult-to-root, 10 genotypes; Supplementary Table S1). Of each of the 15 genotypes, 15–20 shoots were rooted *in vitro* for 10 d on +IBA medium, before the shoots were collected and their base was split into two halves. One half of the samples was frozen immediately in liquid nitrogen, freeze-dried for 2 d, pulverized at 27 Hz for 2 min with two steel beads using a Mixer Mill MM400 (Retsch, Haan, Germany), and analysed by attenuated total reflection (ATR) spectroscopy, as described earlier (Muszynska *et al.*, 2021). In the case of genotypes HF and MT, the second half of the vertically cut shoot bases was used for FTIR imaging.

For data analysis, the OPUS files were imported into MatLab (R2018a, The MathWorks). Spectral data were reduced to a desired spectral range (wave number) of 1800–800 cm^-1^. Vector normalization of the data was carried out, and chemical components were detected using an adapted EMSC (Extended Multiplicative Signal Correction) model (Bassan *et al.*, 2012; Gündel *et al.*, 2018, 2021), including the spectral features of holocelluloses and lignin by partial least squares regression. Principal component analysis was conducted with vector-normalized spectral data of samples. Relative absorbance for IR components was calculated by setting the compound’s absorbance in relation to the total absorbance. Pearson’s correlation coefficients were calculated for mean relative compound absorbance and AR formation percentages, after 10 and 21 d on +IBA, and 21 d on –IBA. To analyse the relationship between rooting ability and the relative absorbance, non-parametric Mann-Whitney test was performed in case relative percent difference value was >3, and Pearson’s correlation coefficients were significant (*P*<0.05) with at least two out of three AR formation percentages.

### Spatially resolved Fourier-transformed infrared spectroscopy in tissue sections

For FTIR imaging, tissues were cut into 8 µm sections using a rotating microtome (Hydrax M55, Zeiss, Oberkochen, Germany), and then transferred onto RNAse-free MMI membrane slides (Molecular Machines & Industries, Eching, Germany). The cross-sections were stored in darkness at 20 °C until FTIR measurement. Adjacent RAA-stained cross-sections were used to define representative regions for analysis via spatial FTIR. FTIR imaging and data analysis were performed according to Gündel *et al.* (2021). Imaging acquisition was performed using a Hyperion 3000 FTIR microscope (Bruker Optics) coupled to a Tensor 27 FTIR spectrometer (Bruker Optics) with an internal mid-infrared source. The focal plane array detector (64 × 64 pixels) was used in transmission mode. The imaging system was continuously purged with dry air. FTIR images were recorded in the spectral range of 3900 to 800 cm^−1^ at a spatial resolution of 11 μm and a spectral resolution of 6 cm^−1^ using 3.5× (15× for high detail images; 5.5 μm digital resolution with 2 × 2 pixels binning) infrared magnification objectives (Bruker Optics). Each spectrum comprised 64 co-added scans. A reference of a single focal plane array window of the empty light path was acquired before image acquisition and automatically subtracted from the recorded image using OPUS software (Bruker Optics). Atmospheric absorptions of water vapour and CO_2_ were corrected by OPUS during image acquisition. OPUS files were imported into MatLab (MathWorks) as ENVI files using the multiband-read function or the irootLab toolbox (Trevisan *et al.*, 2013). Spectral features such as structural polymer fingerprints, along with baseline features, were extracted using an EMSC model adopted into an in-house developed analytical MatLab routine for statistical and quantitative spectral feature analysis as described by Gündel *et al.* (2018, 2021). As paraffin embedding purges solutes from the tissue sections, only cell wall and membrane-bound components remained (lipids, lignin, holocelluloses, proteins, paraffin and other polymers). Mann–Whitney test (*P*<0.05) was used to compare IR traits to account for non-normal distribution of image data. FTIR parameters were further investigated towards their effect size (Cohen, 1962; Fritz *et al.*, 2012) to support the significance claim of the statistical test. The following effect size thresholds were set: small effect: *D*>0.2; medium effect: *D*>0.5 and strong effect: *D*>0.8. Pearson’s correlation coefficients were calculated to investigate the degree of linear dependence between traits.

### Immunolabelling of cell wall compounds

Stem base cross-sections corresponding to those imaged with FTIR of genotypes HF and MT were mounted onto glass slides and used for cell wall immunolabelling using an adjusted protocol by Avci *et al.* (2012). Dewaxed and rehydrated cross-sections were circled on the glass slide with a hydrophobic Super PapPen (Science Services GmbH, Munich, Germany) and incubated for 30 min in phosphate-buffered saline (PBS) with 3% bovine serum albumin (BSA, w/v) followed by three 5 min washes with PBS. Next, cross-sections were incubated with a 5-fold dilution of one of the following monoclonal antibodies (mAbs) in PBS (pH 7) with 3% BSA (w/v) for 1 h: LM6 (anti-arabinan), LM11 (anti-xylan/arabinoxylan), LM21 (anti-mannan), and LM25 (anti-xyloglucan); all mAbs were obtained from PlantProbes (Leeds, UK). A negative control was incubated in PBS with 3% BSA (w/v). Prior to the immunolabelling procedure with mAb LM21, these cross-sections were incubated in 0.1 M sodium carbonate (Na_2_CO_3_, pH 11.4) for 2 h and washed twice in PBS for 10 min each, followed by 2 h in 30 U ml^–1^ pectate lyase (*Aspergillus* sp., EC Number 4.2.2.2, Megazyme Ltd., Wicklow, Ireland) in 50 mM CAPS buffer (pH 10; Marcus *et al.*, 2010; Kim and Daniel, 2017). Pre-treatment prior to labelling with mAb LM21 was ended with two washing steps in deionized water for 5 min each. Subsequent to the immunolabelling with mAbs, the cross-sections were washed three times for 5 min each in PBS, before incubation with a 100-fold dilution of the secondary fluorescence antibody Alexa Fluor 488 anti-rat (Thermo Fisher Scientific Inc., Waltham, MA, USA) in PBS for 1.5 h in darkness, followed by three final washing steps in PBS for 5 min each. Prior to microscopy, cross-sections were incubated for 2 min in 0.05% toluidine blue solution to minimize autofluorescence and rinsed in deionized water. Fluorescence microscopy was carried out using a fluorescence microscope (Axioscope A1; Filterset 38s 470–440 nm excitation, 525–550 nm emission; Zeiss, Jena, Germany) with an exposure time of 400 ms at 200× magnification; light microscopy images were taken of the same regions for every cross-section. Fluorescence intensity was determined for the distinct clusters 2–4 resulting from hierarchical clustering of FTIR results using ImageJ (version 2.9.0/1.53t; Rueden *et al.*, 2017). Fluorescence microscopy images (green channel) and light microscopy images (8-bit) were merged to quantify fluorescence intensity in the green channel. Clusters were selected by hand. For a detailed workflow of fluorescence intensity detection, the reader is referred to Supplementary Fig. S2.

Determined fluorescence intensities were analysed using *R* software (v4.2.1, R Core Team, 2021). Intensities for cluster 2 and a combination of clusters 3 and 4 as weighted mean were analysed, taking into account the clusters´ area [pixels^2^]. Differences in fluorescence intensities between genotypes HF and MT were tested by Wilcoxon Rank Sum test, separately for each mAb.

### Detection and tissue-specific evaluation of lignin distribution

Improved Mäule staining to distinguish syringyl-type (S-type) from guaicyl-type (G-type) lignin (Yamashita *et al.,* 2016) was applied to histological cross-sections corresponding to those analysed by spatial FTIR and immunolabelling. Cross-sections were circled on the glass slide with a hydrophobic Super PapPen (Science Services GmbH, Munich, Germany), incubated in 1% (w/v) aqueous KMnO_4_ solution for 1 min, and rinsed three times with deionized H_2_O. Subsequently, air-dried sections were mounted in 1 M Tris-HCl buffer (pH 8) and covered with a cover slip. Cross-sections were microscopically analysed within 15 min, using a dissecting fluorescence microscope (BX-60, Filterset U-MWB filter 450–480 nm excitation, ≥520 nm emission, Olympus Europe, Hamburg, Germany) with an exposure time of 1 s at 200×, and light microscopic images were taken of the same regions. RPs, assessed at various developmental stages, exhibited distinctive fluorescence patterns contingent upon their stage of development. Consequently, the quantities of RPs at different stages were documented: 1, dome-shaped root primordium; 2, root primordium with vascular system; 3, emerged root primordium. The observed fluorescence in the root cap region of primordia of distinct developmental stages was categorized into four patterns: A, full root cap with strong fluorescence; B, whole root cap with low fluorescence; C, only parts of root cap fluorescent; D, no fluorescence. In total, 10 and 13 primordia were analysed for HF and MT, respectively.

## Results

### AR formation characteristics differ between genotypes HF and MT

**Fig. 1.**
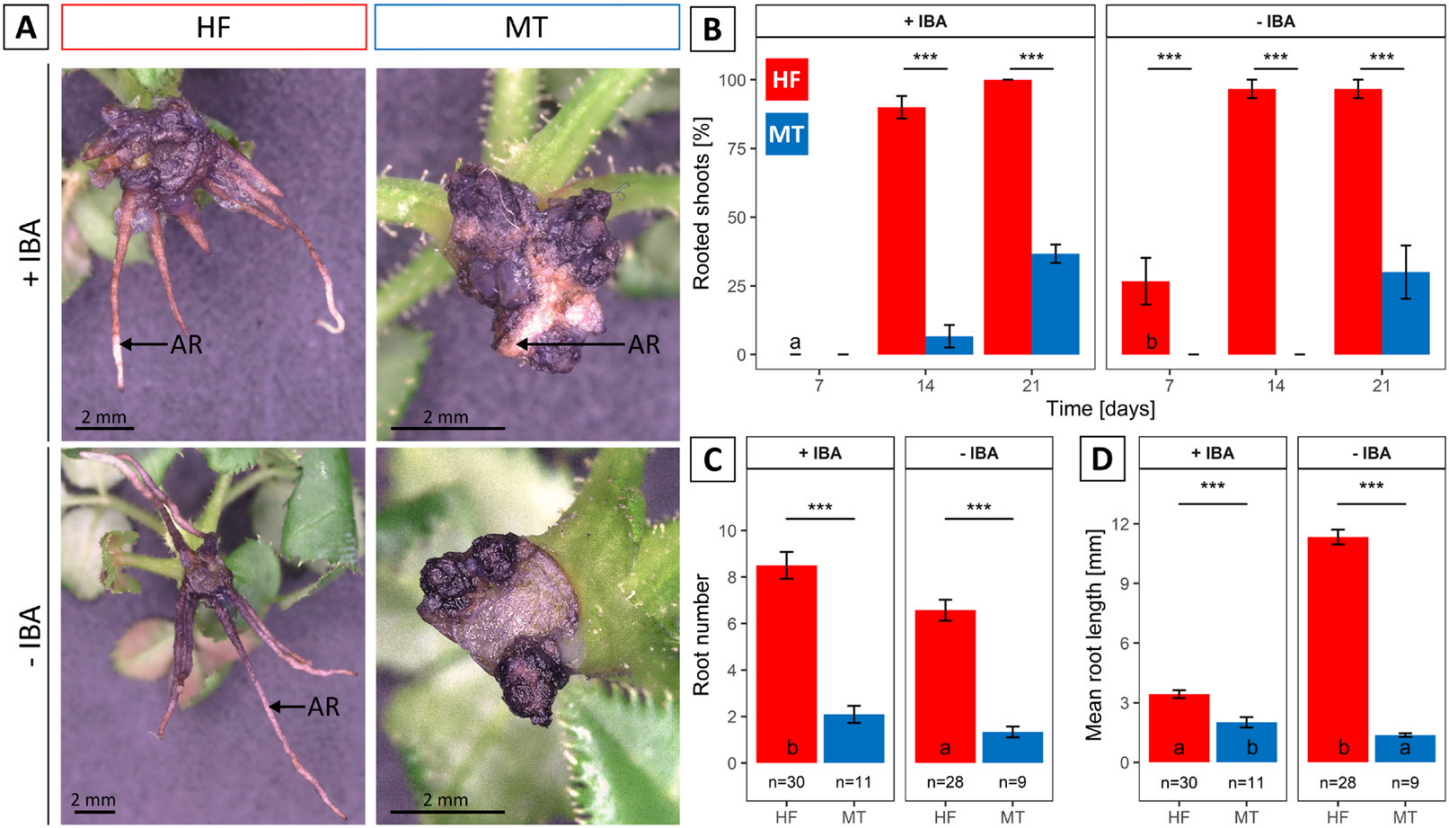
Effect of IBA on AR formation in genotypes HF and MT. (A) Genotype performance after 3 weeks of cultivation on rooting media with (+IBA, 0.98 µM) and without IBA (–IBA). (B) Mean rooting percentages [%] after 7, 14, and 21 d of cultivation for five vessels with six shoots each. (C) Root number per rooted shoot after 21 d of cultivation; *n* displays the number of rooted shoots per genotype and IBA treatment. (D) Mean root length [mm] per rooted shoot after 21 d of cultivation; *n* displays the number of rooted shoots per genotype and IBA treatment. Data of repetition 1 are presented here, while data of repetition 2 are shown in Supplementary Fig. S2. Lowercase letters indicate significant (*P*<0.05, Tukey’s test) differences between the IBA variants within one genotype, while asterisks indicate significant differences between the genotypes within one IBA variant, *P<0.05, **P<0.01, ***P<0.001; Tukey’s test, *α*=0.05. Whiskers indicate SE. AR, adventitious root, indicated by arrows. Scale bars=2 mm.

In order to confirm the contrasting rooting ability and to investigate the effect of IBA on AR formation, shoots of HF and MT were cultivated *in vitro* on a rooting medium with or without IBA. To distinguish them from RPs, we considered only ARs that had emerged from the stem and were clearly visible at the outside of the cutting base. The rooting percentage was evaluated after 7, 14, and 21 d and the root number and root length were measured at the end of the experiment (21 d). Detailed information is presented in Fig. 1, Supplementary Fig. S3, and Supplementary Tables S2, S3. The presence of ARs on a shoot was noticeable as soon as white, juvenile structures separated clearly from the rest of the shoot (Fig. 1). Differences in rooting between HF and MT were visible after 21 d of cultivation on both rooting media (+IBA or –IBA; Fig. 1A). Significant effects for the factors repetition, genotype, time (days of rooting), as well as numerous interactions of different factor combinations were observed (Supplementary Table S2). On +IBA medium, HF showed higher rooting percentages of 100% (21 d) than MT with 36.7% (21 d; Fig. 1B). Likewise, after 21 d on –IBA medium, 96.7% of the HF shoots were rooted compared with 30% for MT (Fig. 1B). Pairwise comparisons between the IBA variants revealed only one significant difference (*P*<0.05, Tukey’s test) for HF at 7 d in rep1, with higher values on –IBA (26.7%) than on +IBA (0%; Fig. 1B).

At the end of the rooting experiment, statistical analysis of the root number per rooted shoot revealed significant effects for the factors genotype and IBA variant (Supplementary Table S2). The average root number differed between genotypes: on +IBA medium, HF formed 8.5 roots per rooted shoot, more than that seen in MT with 2.1 roots (Fig. 1C), and also on –IBA medium, HF shoots formed more roots (6.6) than MT (1.3; Fig. 1C). The values for HF were higher on +IBA medium compared with –IBA medium, while for MT no significant effect of the IBA on root number was recorded (Fig. 1C).

On +IBA medium, HF shoots showed an average root length of 3.4 mm, while roots of MT measured <2 mm (Fig. 1D). On –IBA medium, HF roots were longer, at 11.3 mm, compared with those on +IBA medium and MT roots (1.4 mm; Fig. 1D). ANOVA identified significant effects of the factors genotype and IBA variant, as well as of the interaction of genotype and IBA variant on root length (Supplementary Table S2). Overall, the significant differences between the genotypes in rooting performance were not strongly affected by external application of auxin.

**Fig. 2.**
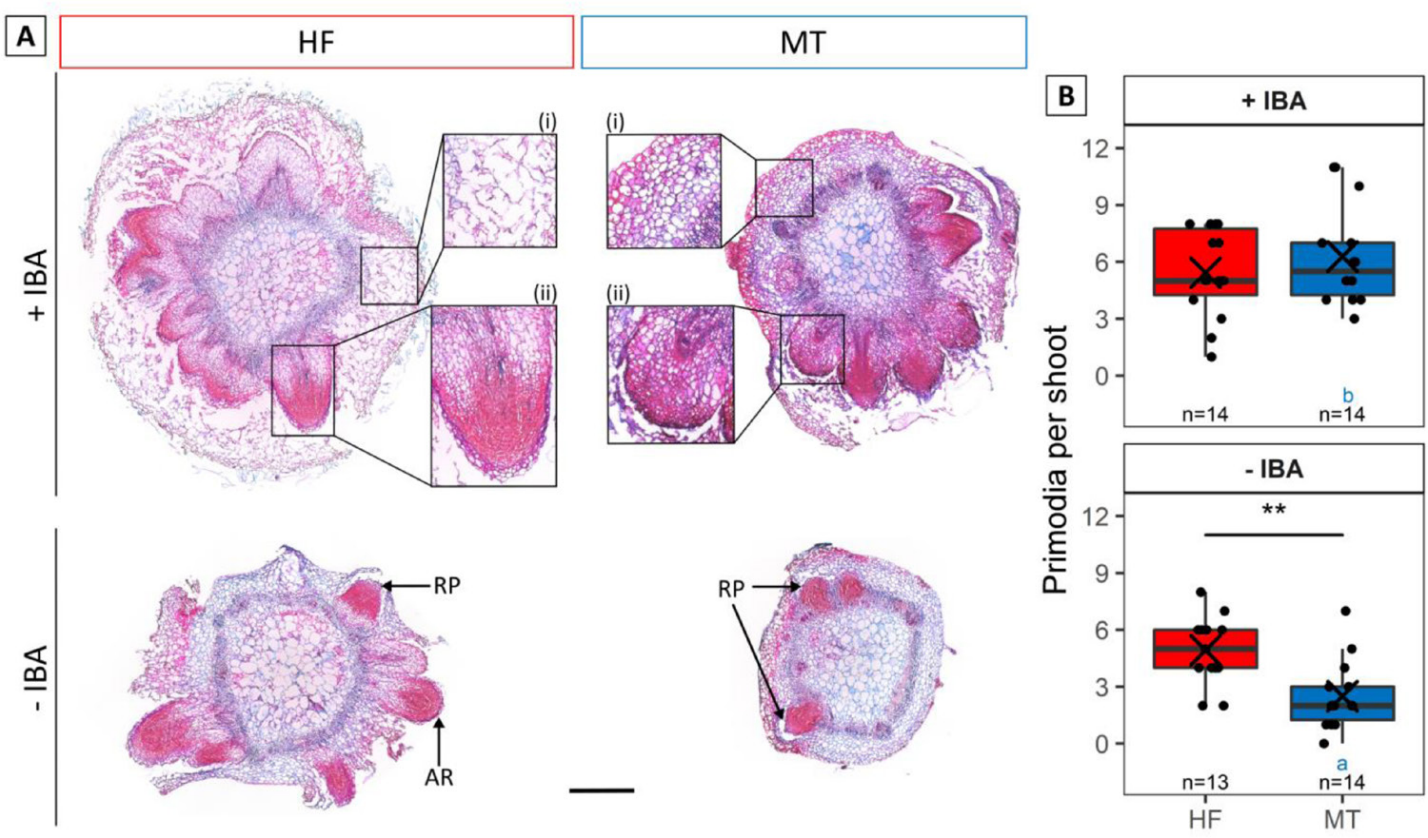
Root primordia (RP) formation in genotypes HF and MT after 7 d of cultivation on rooting medium with (+IBA, 0.98 µM) or without IBA (–IBA). (A) Histological RAA-stained shoot base cross-sections show root primordia (RPs) with (i) detailed section of shoot cortex and (ii) of a RP. Scale bar=500 µm for whole cross-sections and 250 µm for detailed sections. (B) Comparison of RP formation between genotypes HF and MT on rooting medium with (+IBA, 0.98 µM) or without IBA (–IBA); *n* displays the number of analysed cross-sections per genotype and IBA variant, X indicates the mean number of RPs per shoot. Small coloured letters indicate significant differences (*P*<0.05) between the IBA variants within a genotype, while asterisks indicate significant differences between the genotypes within one IBA variant, *P<0.05, **P<0.01, ***P<0.001; Tukey’s test, α=0.05. RP, root primordium.

To investigate whether differences in AR formation between HF and MT were due to differences in RP formation, histological analyses were conducted. The formation of RPs was observed in both HF and MT, on +IBA and –IBA medium (Fig. 2A). The developing RPs of MT appeared more stunted compared with HF. This was reflected in significantly (*P*<0.05, ANOVA) larger values for RP width, length, and area, as well as larger values for the ratio of RP length to RP width (*P*<0.1) for HF (Supplementary Table S4). The cortex tissue seemed to be compact in MT, but looser in HF, as determined by the number of cells per 150 µm, especially when counted in front of a RP (Fig. 2A; Supplementary Table S4). Significant effects (*P*<0.05, Tukey’s test) were recorded in the deviance analysis for the factor IBA variant, and for the interaction of genotype and IBA variant (Supplementary Table S2). On rooting medium with IBA, no significant difference (*P*<0.05, Tukey’s test) in RP number per shoot was noticeable between HF (5.4) and MT (6.3; Fig. 2B). However, on medium lacking IBA, the superior rooting response of HF was reflected by its significantly higher (*P*<0.05, Tukey’s test) number of 4.9 RPs compared with MT, with 2.5. Comparison of IBA variants separated by genotype showed significantly higher (*P*<0.05, Tukey’s test) RP numbers for +IBA compared with –IBA only for genotype MT (Fig. 2B). Thus, IBA treatment led to an increase in the number of RPs per shoot in the difficult-to-root genotype MT, but not in the easy-to-root genotype HF.

### Magnetic resonance imaging-enabled non-invasive 3D investigation of adventitious root formation in genotypes HF and MT

**Fig. 3.**
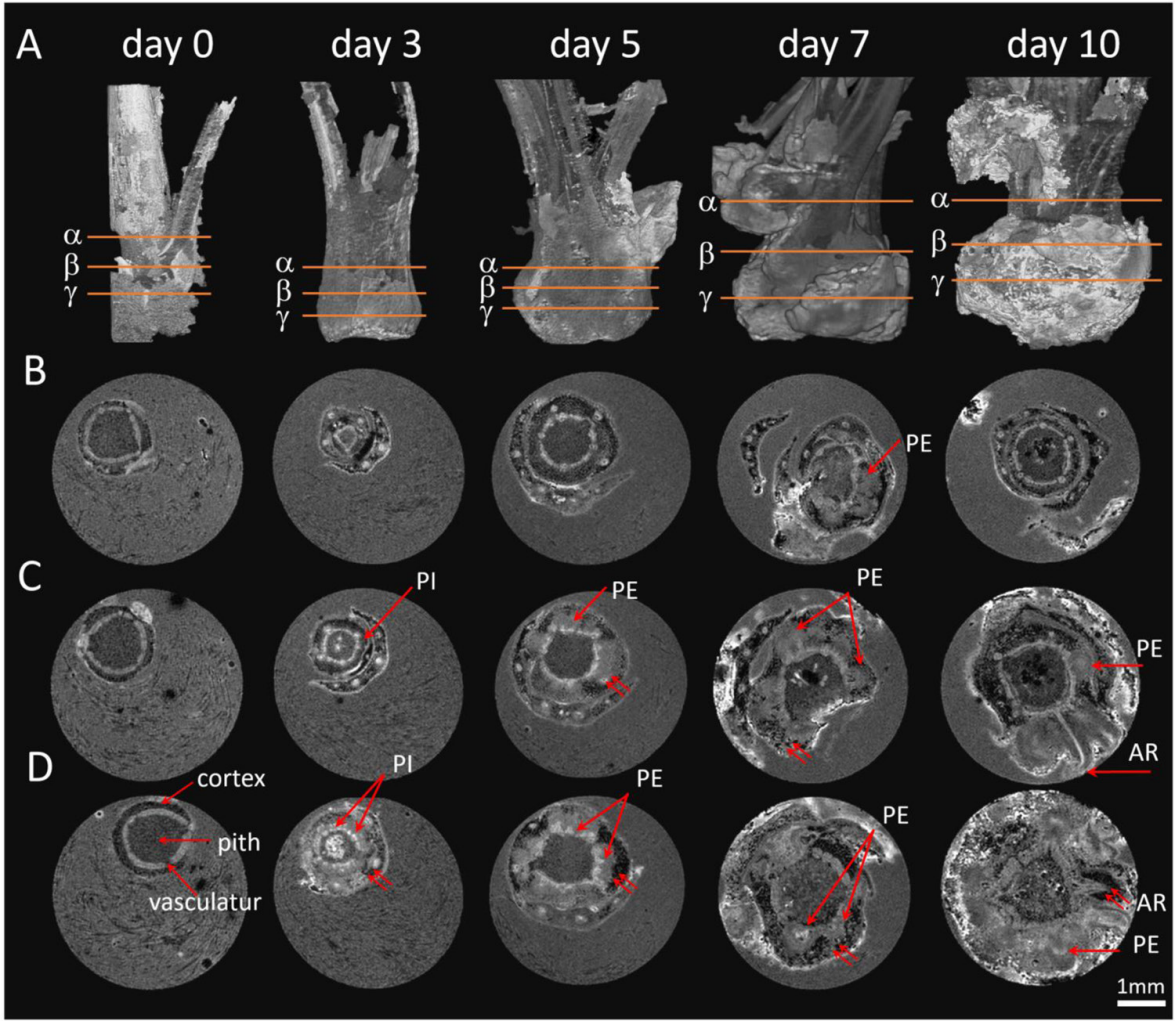
NMR imaging of root primordia formation in genotype HF. (A) The stem structure of HF cuttings in 3D after 0, 3, 5, 7, and 10 d of cultivation on rooting medium with IBA (0.98 µM) (see also Supplementary Video S1). (B-D) Virtual cross-sections of the cuttings at the upper (α), middle (β) and lower levels (γ). AR, adventitious root; PE, primordia elongation; PI, primordia initiation. Scale bar=1 mm.

**Fig. 4.**
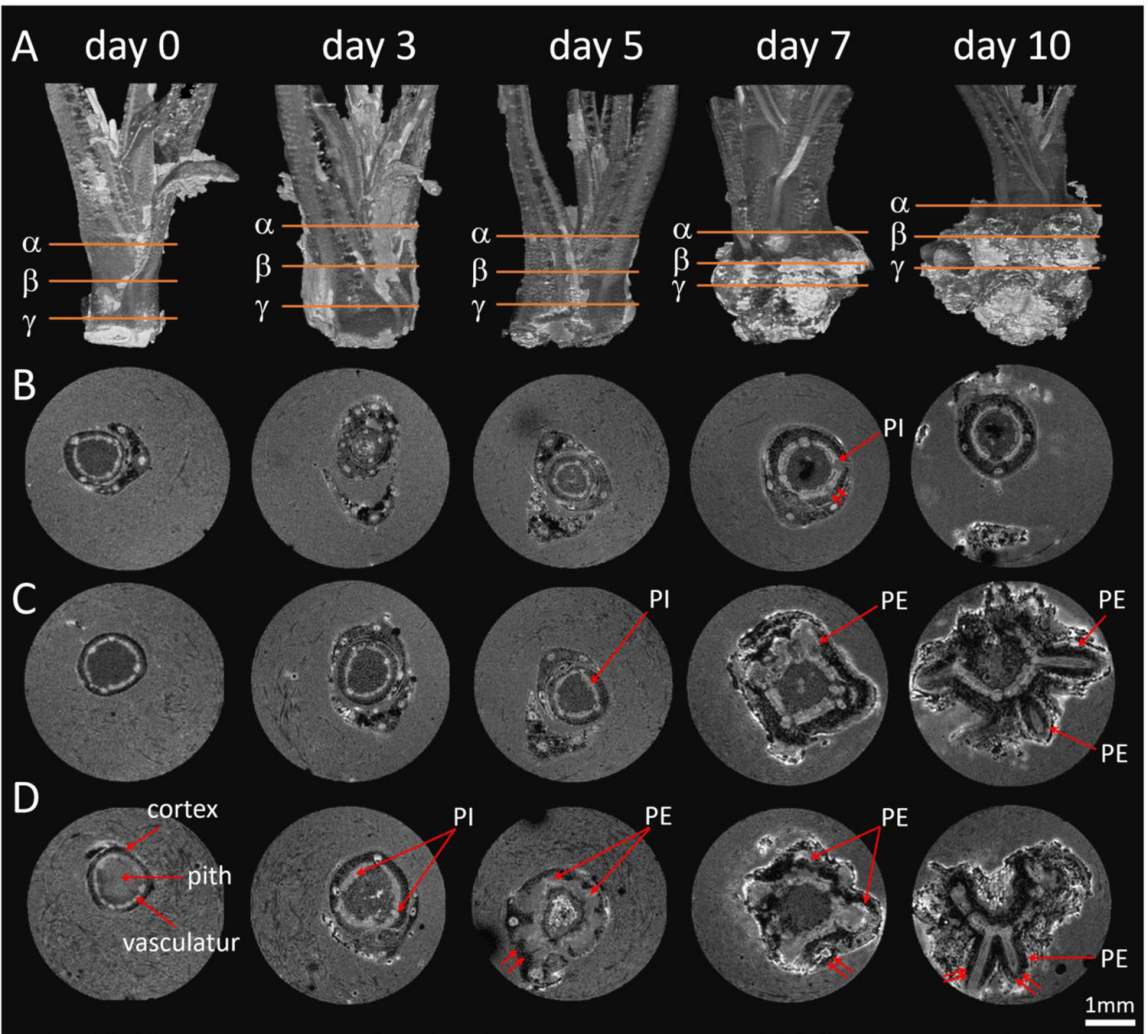
NMR imaging of root primordia formation in genotype MT. (A) The stem structure of MT cuttings in 3D after 0, 3, 5, 7, and 10 d of cultivation on rooting medium with IBA (0.98 µM) (see also Supplementary Video S2). (B-D) Virtual cross-sections of the cuttings at the upper (α), middle (β) and lower levels (γ). AR, adventitious root; PE, primordia elongation; PI, primordia initiation. Scale bar=1 mm.

During the rooting experiment, we observed a characteristic thickening in the lower part of the stem in both genotypes. This thickening culminated in the emergence of roots in HF, but not in MT. To investigate the inner architecture of the swollen part of stem and to determine the structural differences between the two genotypes, we used non-invasive 3D MRI. The main tissues of stems, such as pith, vascular ring, cortex and RPs were well defined in the 3D images (Supplementary Videos S1, S2). Histological analysis of the same specimens using light microscopy justified our tissue identifications (Fig. 2A). According to the comparative 3D MRI of the two genotypes (Figs 3, 4), the lower part of the stem contained a large number of RPs that were hidden inside. However, few, if any, had penetrated the surface of the stem to become visible and identifiable as ARs in MT (Fig. 4).

We characterized the developmental process of AR formation in more detail: in the early stages of cultivation, the MRI of the stem of the HF cuttings showed a well-organized pattern with a bright inner ring of vasculature. The vasculature was bordered on the inside by pith tissue and on the outside by cortex tissue, which were well distinguished in greyscale (Fig. 3B–D, day 0). After the first 3 d of incubation, the tissue pattern of cuttings became less regular, as evidenced by the brightening of the MRI signal in regions corresponding to the vasculature and other tissues associated with the vascular ring (Fig. 3B–D, day 3). After 5 d, some of these regions became distinct from both the vasculature and the cortex parenchyma due to the altered proton density (Fig. 3C, D, day 5). Formation of such a structure (primordium) was observed only in the basal/lower part of the cuttings and followed by their asymmetric increase in diameter. Further on, they became bell-shaped and continued to elongate in a centrifugal direction (Fig. 3C, D, day 7). At this stage, the elongation process was accompanied by structural changes inside the RPs. We observed an increase of tissue density in their peripheral versus central part, and finally their protrusion through the surface of the cuttings (Fig. 3C, D, day 10). Therefore, the tissues of the vascular ring and the central regions of RPs were continuous and exhibited similar proton densities, indicating a connection. At this point, these structures were clearly identified as ARs. Thus, in the easy-to-root genotype HF, ARs originated in the primary vascular bundles and grew out through the cortex within 7–10 d.

While similar events took place in the genotype MT during the early and middle stages of AR formation (Fig. 4A) and a large number of RPs were formed, the characteristics of the tissue structures were different from those of the genotype HF (Fig. 4B-D). For none of the RPs did we observe such a pronounced elongation, that it led to an emergence and development of an AR, as observed for HF (Fig. 4B-D). Thus, in the difficult-to-root genotype MT, new RPs developed from the primary vascular bundles, but failed to emerge through the outer layer of the stem and to elongate into an AR.

### Contrasting phenotypes of AR formation are a consequence of histological differences between genotypes HF and MT

**Fig. 5.**
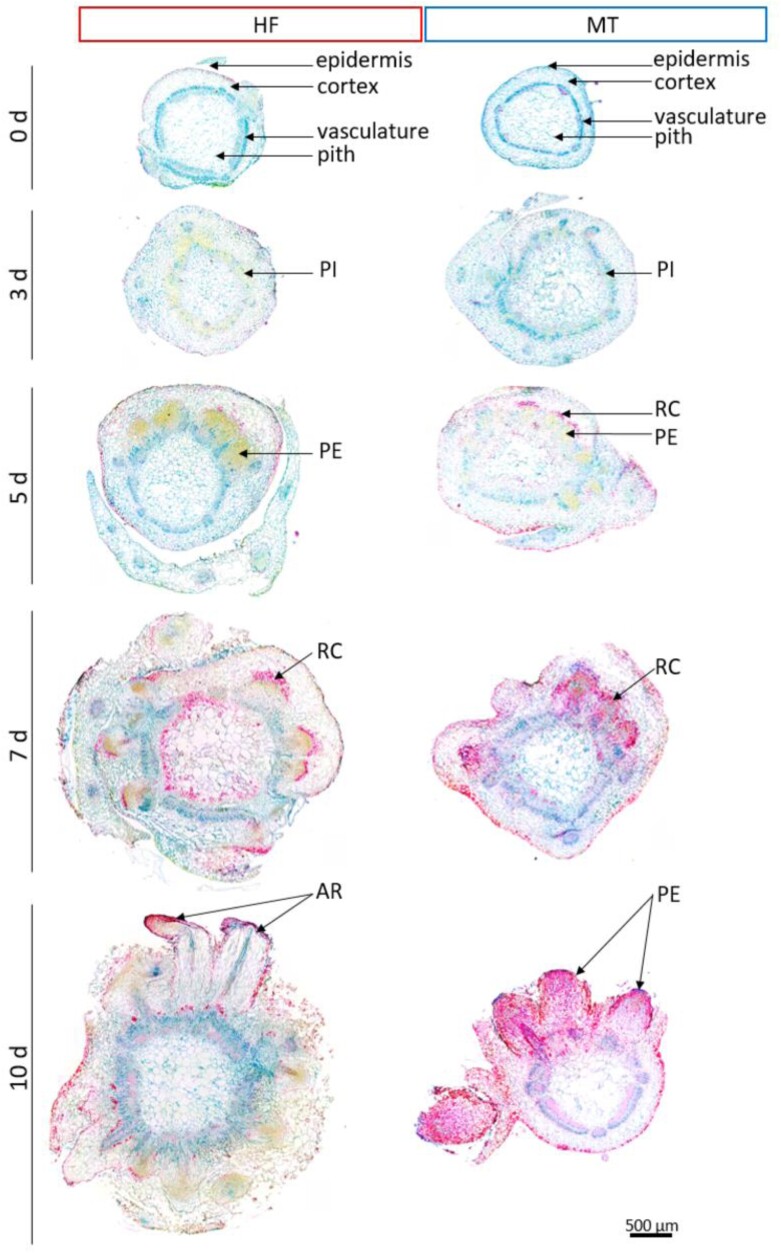
Histological analysis of NMR-imaged shoot bases to validate MRI tissue identification and to study root primordia formation. Characteristic shoot base cross-sections of genotype HF and genotype MT imaged and sampled after 0, 3, 5, 7, and 10 d on rooting medium with IBA (0.98 µM), serially stained with RAA. AR, adventitious root; PE, primordia elongation; PI, primordia initiation; RC, root cap. Scale bar=500 µm.

In order to spatially correlate the non-invasive MRI observation with the classical histological study, we targeted the region of interest (detected by non-invasive MRI) by detailed light microscopic analysis at each particular stage of RP formation. Histological images obtained from the same shoots analysed with NMR technique were found to be in good agreement with the MRI findings (Fig. 5). The first changes in internal structures of the stem were observed in the region of vasculature after 3 d (Fig. 5A). After only 5 d of cultivation, both genotypes clearly demonstrated high numbers of RPs, four in MT and seven in HF. These RPs were visible by histological staining in yellow due to binding of acriflavine to nucleic acids in the 2D microsections (Fig. 5, 5 d), which corresponded well with the virtual MRI sections of the shoot (Fig. 3C, D, 5 d; Fig. 4D, 5 d). Elongated RPs were well distinguished due to their dense cellular structure. Intense red colouration by RAA staining in front of the forming RPs showed the formation of the protective root cap for MT after 5 d (Fig. 5B, 5 d), and for HF after 7 d (Fig. 5A, 7 d). In contrast to HF, MT exhibited a red colouration of parenchyma (stem cortex) and RPs by RAA staining that became even stronger at later stages after 7 and 10 d, indicating a more intense accumulation of lignin, carbohydrates or other cell wall-associated compounds (Fig. 5B).

Although thickening in the lower part of the stem was observed in both genotypes, characteristic differences became apparent from day 5. In addition to the changes in colouration, this is also reflected in the size of the cross-sections. Furthermore, the cell structure of the shoot cortex appeared looser and less integrated in HF than in MT (Fig. 2A). Between days 5 and 7, HF showed a greater growth of the vasculature, so that from day 7 the radial expansion was more pronounced than that of MT (Fig. 5; Supplementary Table S5). This led to increased traction in the tissues in front of the developing RPs, namely the shoot cortex. During to the continued intensive growth of the HF RPs, the cell integrity of the shoot cortex was disrupted, and a lower cell density in MT was measured after 7 d (Supplementary Table S5). There was a clear progression of the RPs through the parenchymal shoot cortex in both genotypes after 7 d, but the apex of the RPs in MT remained behind the epidermis of the stem. The RPs of MT appeared more stunted compared with HF, pointing to a possible mechanical resistance of the more solid cortex tissue (Fig. 5).

We hypothesized that the contrasting rooting behaviour of HF and MT may be related to differences in the chemical tissue environment (e.g. cellular structure, composition, cell wall rigidity), and is manifested during the elongation and emergence of ARs.

### Fourier transformation infrared spectroscopy revealed characteristic differences between easy- and difficult-to-root genotypes

To address the question of possible biochemical differences between genotypes that exhibit different AR-formation phenotypes, we selected 15 different rose genotypes for further experiments based on their distinct ability to form ARs. These were classified into categories of easy-to-root and difficult-to-root genotypes (Supplementary Table S1). The biochemical composition of the shoot bases was determined by ATR-FTIR spectroscopy, which documented clear differences in the tissue composition of the contrasting groups, and correlations to rooting percentages after 10 and 21 d of cultivation on (+IBA) rooting medium (Supplementary Table S6). In particular, we identified compositional differences of MT and HF under different IBA treatments (Supplementary Table S6).

Good predictive performances could be detected for 17 single compounds and 18 compound groups. For six of these single compounds, Pearson’s correlation coefficients with AR formation percentages after 10 and 21 d on +IBA and after 21 d on –IBA were significant (Supplementary Table S6). Analysing the distinctions between genotypes that are easy to root and those that are difficult to root in relation to the six individual compounds revealed significant differences for five out of the six compounds (Supplementary Fig. S4). The group of easy-to-root genotypes showed lower relative levels for the amino acid alanine (Ala), 3-phosphoglyceric acid, and the ethylene precursor 1-aminocyclopropane-1-carboxylic acid (ACC), while values were higher for the cytokinin precursor N6-isopentenyl-adenosine and fructose (Supplementary Fig. S4). For *t*-ferulic acid, the correlations were significant for all three AR formation traits, but the easy-to-root and difficult-to-root genotype groups were not statistically different from each other (Supplementary Fig. S4).

**Fig. 6.**
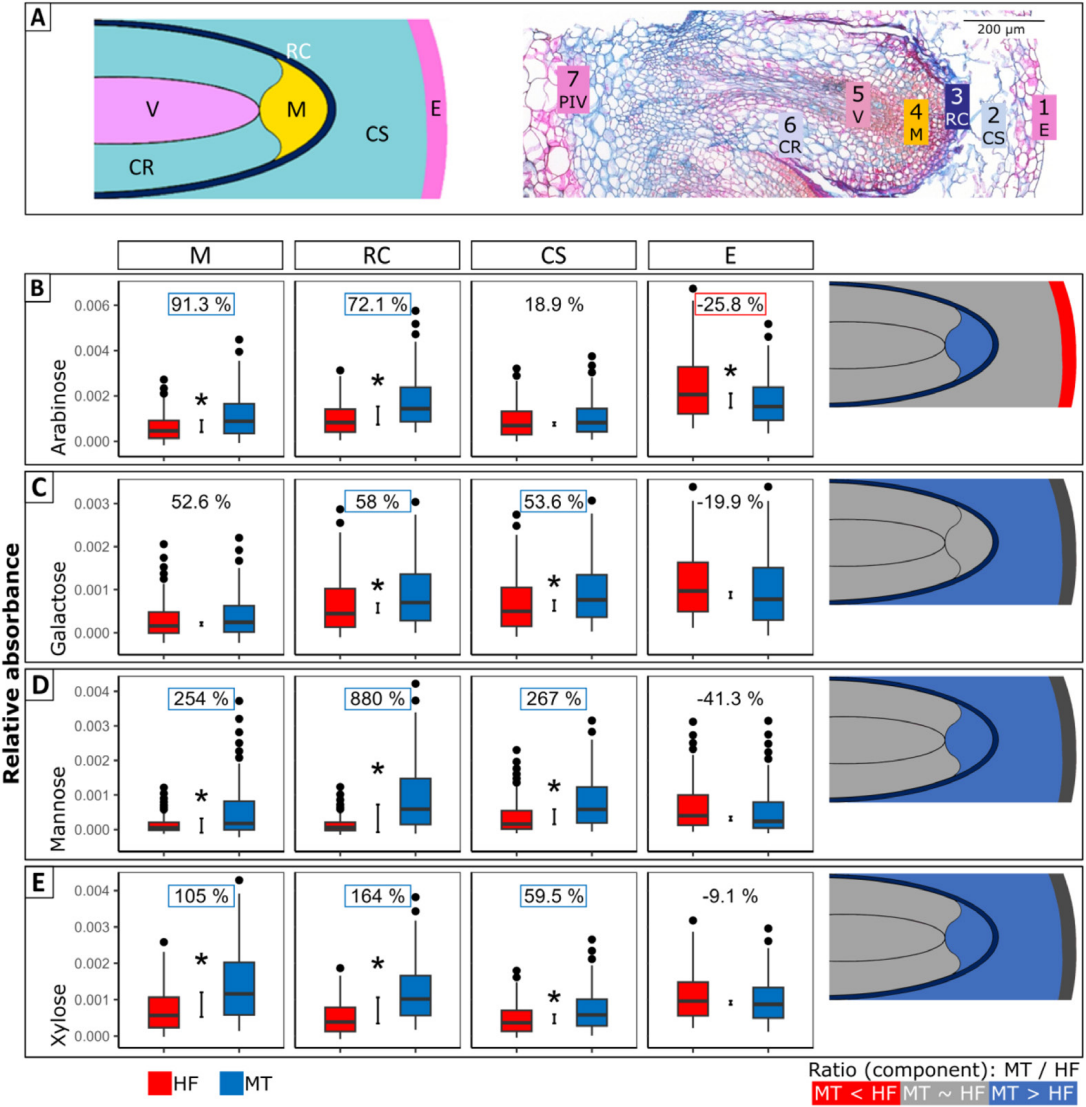
Characteristic FTIR spectra fingerprint clusters and cell wall compounds detected in cross-sections of genotypes HF and MT. (A) Scheme of the characteristic seven (1–7) clusters within a rose shoot base cross-section generated by hierarchical clustering of fingerprints (left) and localization in a cross-section of genotype MT after 10 d on +IBA medium; representing functional tissues of: 1 E, epidermis; 2 CS, cortex shoot; 3 RC, root cap; 4 M, meristematic region; 5 V, vasculature; 6 CR, cortex root; 7 PIV, pith and vascular ring. Scale bar=200 µm. (B–E) Results for (B) arabinose, (C) galactose, (D) mannose, and (E) xylose for the clusters 1 to 4 in genotypes HF and MT, representing M, RC, CS and E, respectively, from left to right. Boxplot graphs show median values as thick black horizontal lines, interquartile range (IQR) as coloured box, and whiskers representing 1.5 × IQR; error bars show the quantile absolute difference (QAD); * indicates significant difference between the two distributions (Mann-Whitney *P*<0.001) and the divergence effect size was >0.8, or *R*^*2*^ of the divergence effect size was >0.5. Colouring in cluster in schemes indicates significant differences between the two tested genotypes within the single clusters. Grey colour indicates no difference, red colour indicates lower and blue colour higher values for MT compared with HF. The percentage values indicate the ratio of the difference between the values of MT and HF (MT–HF) in relation to the value for HF taken as 100%.

To compare the composition of tissues involved in AR formation and those surrounding the developing RPs in the different genotypes, we used spatially resolved FTIR in shoot base cross-sections for genotypes MT and HF. Hierarchical clustering on FTIR spectra fingerprints of tissue sections revealed seven clusters that can be assigned to characteristic tissue regions of epidermis (cluster 1), shoot cortex (cluster 2), root cap (cluster 3), AR meristematic tip (cluster 4), AR vasculature system (cluster 5), AR cortex (cluster 6), and pith and vascular ring of the shoot (cluster 7; Fig. 6A). Tissues instantly interacting during elongation and outgrowth of AR demonstrated differences in cell wall composition between HF and MT (Fig. 6B–E). In particular, the stem epidermis of easy-to-root HF was characterized by the relatively higher absorbance of the hemicelluloses associated with arabinose (Ara, Fig. 6B) monomers present in hemicellulosic polymers, whereas galactose (Gal, Fig. 6C), mannose (Man, Fig. 6D), and xylose (Xyl, Fig. 6E) monomers present in hemicellulosic polymers were lower in the stem cortex of HF. The AR apex of difficult-to-root MT demonstrated higher absorbance of Ara (Fig. 6B), Man (Fig. 6D), and Xyl (Fig. 6E). For all of the four hemicellulose subunits, significantly higher values (1. *P*<0.001, Mann-Whitney test and 2. divergence effect size >0.8 or *R*^*2*^_divergence effect size_>0.5) were found in the root cap (cluster 3) of MT compared with HF, with highest differences for Man- and Xyl-hemicellulose signatures (Fig. 6B–E). In the shoot cortex (cluster 2) higher values could be detected for Gal, Man, and Xyl in MT compared with HF (Fig. 6C–E). Higher values for MT than for HF in the AR meristematic tip (cluster 4) were observed for Ara, Man, and Xyl (Fig. 6B, D, E). Values for the additional four clusters can be found in Supplementary Table S7.

Differences in relative abundance between MT and HF for cell wall-related carbohydrates and lignin types (Supplementary Fig. S5) showed up mainly in the shoot cortex and root cap. Lignin was more abundant in MT than in HF, especially in cluster 3 (root cap; Supplementary Fig. S5D), whereas the G-rich lignin was more abundant in the root cap of HF (Supplementary Fig. S5E). In contrast, lignin rich in 5-hydroxyguaiacyl (OHG-rich) was detected in higher quantities in the cortex of MT (Supplementary Fig. S5F). For pectins (rhamnogalacturonan, GalARha) (Supplementary Fig. S5G) the concentration was 77% higher in MT in the root cap (cluster 3). Additionally, differences in protein content and composition between MT and HF were found (Supplementary Fig. S6). The detected total protein content was higher in MT ARs (clusters 4–6) and root caps (cluster 3), and also protein type 1 was observed in higher levels in clusters 1–6 of MT cross-sections. In contrast, protein type 2 was significantly higher (1. *P*<0.001, Mann-Whitney test and 2. divergence effect size >0.8 or *R*^*2*^_divergence effect size_>0.5) in relative abundance in the root cap and cortex within HF stems (Supplementary Fig. S6). The main difference between both protein groups is associated with the relative higher representation of C-O stretching vibrations in type 1 over type 2 spectra. Values for the additional four clusters can be found for cell wall carbohydrates and lignin in Supplementary Table S8, and for proteins in Supplementary Table S9.

Overall, FTIR spectroscopic analysis indicated a pronounced difference between HF and MT in stem tissue composition. According to high resolution FTIR mapping, the tissues directly interacting with the outgrowing ARs were characterized by different cell wall compositions in the two contrasting genotypes.

### Peculiarities of cell walls during AR primordia formation in easy- and difficult-to root genotypes

**Fig. 7.**
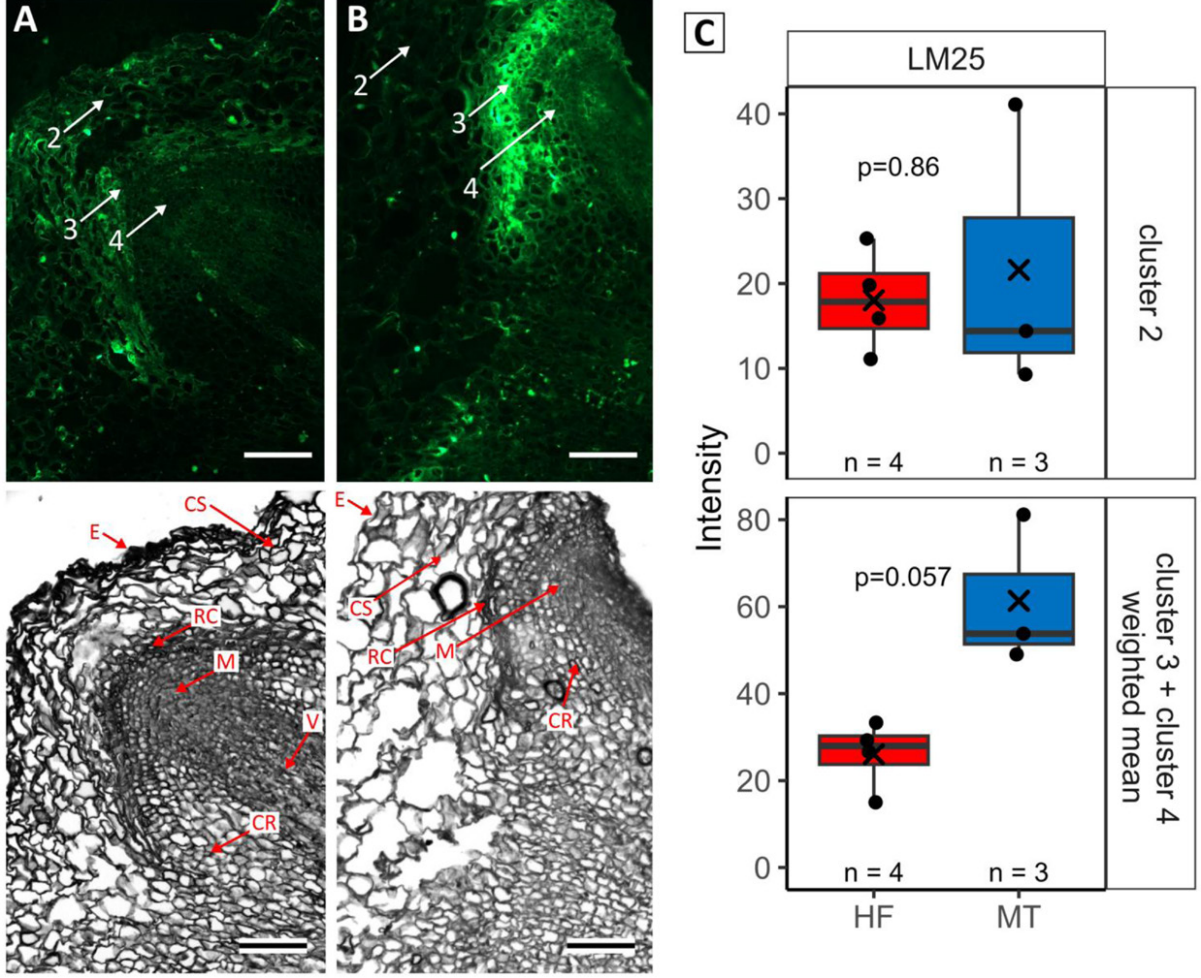
Immunolocalization of xyloglucan in cross-sections of the difficult-to-root genotype MT and the easy-to-root genotype HF. (A) Cross-section through the shoot base with a root primordium (RP) of HF (lower panel) and under fluorescent light after immunostaining with mAb LM25 (upper panel). (B) Cross-section through the shoot base with a RP of MT (lower panel) and under fluorescent light after immunostaining with mAb LM25 (upper panel). Scale bars=100 µm. (C) Quantified fluorescence intensities in the pre-defined clusters 2–4 (see Fig. 6) for genotypes HF and MT and LM25. Mean fluorescence intensities for cluster 2 and combined for clusters 3 and 4 are displayed. The number of analysed RPs per genotype is indicated by *n* and X indicates the mean values. *P*-values are given for results of Wilcoxon rank sum test. The images in (A) and (B) are also shown in Supplementary Fig. S7A for comparison with other antibodies. CS, cortex shoot; CR, cortex root; E, epidermis; M, meristematic region; RC, root cap; V, vasculature.

The secondary cell wall comprises a sophisticated composition of cellulose, hemicellulose (mainly xylan and glucomannan) and lignin. Since some of these compounds were identified to distinguish the two genotypes in FTIR imaging, we continued to define the peculiarities of cell walls of tissues in and directly adjacent to the developing ARs by immunostaining in cross-sections of stem bases after 7 d on +IBA rooting medium. The FTIR spatial results allowed the selection of antibodies to the most promising candidates. Correspondingly, we applied antibodies specifically binding to hemicellulosic epitopes, namely LM11 (anti-xylan/arabinoxylan), LM21 (anti-mannan), and LM25 (anti-xyloglucan), or to pectic epitopes, namely LM6 (anti-arabinan), to cross-sections adjacent to those imaged via FTIR (Fig. 7; Supplementary Fig. S7). The fluorescence intensities for antibodies LM6, LM11, and LM21 were comparatively low (Supplementary Fig. S7), and no significant differences in fluorescence intensity between HF and MT were detectable. In contrast, high average fluorescence intensities were recorded for LM25, indicating xyloglucan in both, the tissues of shoot cortex (cluster 2) and clusters of root cap and root meristem (cluster 3 + 4; Fig. 7B). While intensities in the shoot cortex were similar for both genotypes, within the root cap and root tip, which were analysed collectively, MT showed almost twice as high relative intensity as HF (Fig. 7B).

The mechanical strength and waterproofing of plant secondary cell walls is largely influenced by lignin. We localized lignin in tissue sections using an improved Mäule method (Yamashita *et al.*, 2016), which distinguished between S- and G-type lignin in general, but was not able to detect OHG-type lignin in particular. The labelling co-localized with G-type lignin in the shoot cortex (cluster 2) and especially the root cap (cluster 3; Supplementary Fig. S8B). For HF, 70% of the RPs were in the early developmental stage 1, while for MT only 46% of all primordia were in this developmental stage. This early developmental stage was the only one where no lignin fluorescence signals were recorded in the root cap, and this was observed more frequently in HF (pattern D, HF 40%, MT 8%; Supplementary Fig. S8A). In contrast, RPs showing the most intense fluorescence pattern A were only observed in MT (stage 1: 8%, stage 2: 8%; Supplementary Fig. S8A), indicating stronger lignification of cell walls in this difficult-to-root genotype. This may indicate a higher mechanical resistance to AR outgrowth.

## Discussion

### Exogenous IBA application stimulates root primordia development but not AR formation in the difficult-to-root genotype MT

Enhancement of AR formation by addition of exogenous auxin to the rooting medium has been documented in numerous species (Fattorini *et al.*, 2017; Bai *et al.*, 2020). In our experiment, no improvement in rooting percentage was found by adding exogenous IBA to the difficult-to-root genotype MT. Nevertheless, the number of RPs of MT was significantly increased by the addition of IBA (Fig. 2B). Furthermore, in the absence of exogenous IBA, the easy-to-root HF genotype showed significantly higher root number and length (Fig. 1C, D). This is in accordance with the previously described inhibitory effect of IBA in the later expression phase of AR formation (Pierik, 1997; De Klerk *et al.*, 1999; Druege *et al.*, 2016; Bai *et al.*, 2020).

Various temporal and spatial aspects of auxin supply (e.g. auxin pulses) were addressed in previous studies, and peculiarities in the reaction of distinct genotypes were included (De Klerk *et al.*, 1995; Pierik, 1997; Bai *et al.*, 2020). It can be assumed that the different genotypic responses to auxin supplementation emphasize the complex physiology of AR formation. Our experimental data, both from the histological studies and non-invasive MRI on the two contrasting genotypes HF and MT under different experimental conditions, leave no doubt that exogenous IBA supply did not increase the rooting percentage of the difficult-to-root genotype MT, but may affect signalling cascades leading to a divergence in the rooting response or developmental speed. We conclude that the availability of auxin was not the limiting factor for AR formation in the difficult-to-root rose genotype.

### The distinct properties of cell walls coincide with the differences in rooting ability in various rose genotypes

Spatial FTIR analyses of shoot base cross-sections were performed for HF and MT to approximate the specificity of their biochemical composition. Substantial differences between genotypes were found in the relative spectral absorptions for several cell wall-associated substances and especially cell wall carbohydrates (hemicelluloses) and lignin. Cell walls are usually made up of cellulose, hemicellulose, and pectin (Cosgrove, 1997), and lignification is a major contributor to mechanical strength at later developmental stages (Boerjan *et al.*, 2003; Emonet and Hay, 2022). Our study demonstrates that the easy- and difficult-to-root genotypes have characteristic patterns of hemicellulose and lignin in the tissues directly involved in AR formation, namely shoot cortex (cluster 2), root cap (cluster 3), and root tip (cluster 4; Figs. 6, 7; Supplementary Figs. S5, S8). In particular, the monomeric composition of hemicelluloses, i.e. abundance of Ara, Gal, Man, and Xyl-subunits (Fig. 6B–E), was different in the difficult-to root genotype MT. Hemicelluloses form a heterogeneous group of cell wall carbohydrates with divergent backbones of xyloglucan, mannans, xylose, glucuronoarabinoxylans, or glucans (Duman *et al.*, 2020). By creating a linkage of cellulose fibrils, hemicelluloses influence the elasticity and stiffness of cell walls (Wei *et al.*, 2019). Testing of several mAbs that specifically bind to hemicellulose carbohydrate polymers revealed a notable difference in mean fluorescence intensity for the hemicellulose xylose monomer (LM25), with the difficult-to-root genotype MT having twice as high intensities as HF in the root cap and the root meristem (Fig. 7). Unfortunately, not all cell wall compounds could easily be detected by immunofluorescence, but this does not necessarily mean that the substances are not present (Marcus *et al.*, 2010; Hervé *et al.*, 2011; Verhertbruggen *et al.*, 2017). In particular, the detection of heteromannans via mAb LM21 can be masked either by esterification and/or acetylation in secondary cell walls, or by pectic homogalacturonans (HG) in primary cell walls (Marcus *et al.*, 2010). In further studies a larger sample size is recommended, but in this study, we aimed to immunolabel identical shoot bases, which were also analysed by spatial FTIR.

The amount of lignin was different in the region of the root cap (cluster 3) and in the region of the shoot cortex (cluster 2; Supplementary Fig. S5D–F). Lignin impregnation confers mechanical strength, rigidity, and hydrophobicity (Gibson, 2012; Dixon and Barros, 2019). While lignin content in general in the root cap and OHG-rich lignin (aliphatic) in the shoot cortex was higher in HF compared with MT, the difficult-to-root genotype MT showed higher content of G-rich lignin in the shoot cortex. Furthermore, using modified Mäule staining, we detected G-type lignin in the region of the root cap (cluster 3) and weakly in the region of the shoot cortex (cluster 2; Supplementary Fig. S8B). Only a few dome-shaped early primordia did not show any lignin fluorescence. The fact that the number of RPs without any fluorescence in the root cap was much higher in HF (40%) than in MT (8%) may indicate a higher lignification/less breakdown in the difficult-to-root genotype MT of cell wall structures resisting the protrusion of RPs (Supplementary Fig. S8A). Both aspects would relatively increase the level of lignin in tissue. The summary of FTIR data and immunolabeling results suggests that during RP formation, the cell wall features of the difficult-to-root genotype MT clearly deviated from that of the easy-to-root HF. The high impact of compounds, which function as a tether and play a key role in the loosening and tightening of cellulose microfibrils, points to the abilities of cells to change their shape within the growth and differentiation zones of RPs. In the stem, the increase in hemicellulose xyloglucan rather contributes to the maintenance of the final shape of mature cells of cortex. The mechanical resistance of the cell walls is also fortified by the increased lignification of the cell wall, which was a characteristic of the difficult-to-root genotype MT. Consistent with this, Syros *et al.* (2004) observed that absolute lignin content was higher in shoot bases of a non-rooting genotype of *Ebenus cretica* than in a rooting genotype, during early initiation. High lignification is often also associated with mature tissues or even dead xylem tissue where cells such as tracheary elements lignify even after programmed cell death to form water conducting vessels. The high lignification could also indicate a progressed cell age and therefore cells are more likely to not be able to grow/elongate as would be necessary for root protrusion. Thus, HF could simply have cells which are not progressed as much in their life cycle, and still capable of facilitating growth/elongation.

The observation that lower concentrations of cell wall-stabilizing intact hemicelluloses were found in the easy-to-root genotypes could be related to a weakening of the cell walls, and thus, the mechanical barriers for primordia expansion within the stem tissue. This, in turn, may explain the facilitated emergence of ARs that we have documented in the easy-to-root genotype. During cell growth and differentiation, the processes of cell wall construction and reconstruction are carried out by specific proteins, which are essential components of plant cell walls (Jamet *et al.*, 2006; Couturier *et al.*, 2013; Zhao *et al.*, 2013). They are involved in protein/protein or protein/polysaccharide interactions that determine the properties of cell walls, signalling, and interactions with plasma membrane proteins at the cell surface (Cannon *et al.*, 2008). Although we documented differences in protein composition between the easy- and the difficult-to-root genotype (Supplementary Fig. S6B–E), further proteomic and molecular studies are required to explore their relevance. In a genome-wide association study on AR formation in roses, Nguyen *et al.* (2020) found two single nucleotide polymorphisms located in *CELLULOSE SYNTHASE-LIKE* (*CSL*) subfamily of genes, both encoding enzymes with mannan synthase activity, to be significantly associated with *in vivo* rooting traits, namely root number and root length. Our study provides evidence that the way in which cell walls are arranged (including their structure, composition, and mechanical properties) has a significant impact on the efficiency of AR formation. This finding is a crucial step towards investigating in greater detail the control mechanisms of AR formation.

The analysis of further genotypes with distinct rooting phenotypes harbours the potential to unravel the possible involvement of further known players in limitation of AR formation in rose. For example, analysis of the pulverized shoot bases of the 15 rose genotypes yielded significantly higher absorbance values for ACC, an ethylene precursor, in easy-to-root genotypes. ACC itself has been shown to promote AR formation in *Arabidopsis thaliana* (Rasmussen *et al.*, 2017). Of particular interest in the context of probably enhanced cell wall loosening in some easy-to-root rose genotypes is ethylene-induced programmed cell death and weakening of epidermal cells, and thus promotion of AR emergence, as described in *Oryza sativa* (Mergemann and Sauter, 2000; Steffens and Sauter, 2005).

### Relevance of magnetic resonance imaging and spatial approaches to study adventitious root formation

Our study of AR formation integrates modern spatial approaches with classical histological methods. Histochemical staining, such as RAA for tissue structure, localization of lignin, or immunostaining, require specific tissue treatment and are destructive to the plant. Although accurate and informative, they are extremely time and labour intensive, especially when comparing the internal structure of relatively large specimens used to evaluate the ARs inside rose microcuttings. It was necessary to prepare a large number of individual explants at different times within a time series. Non-invasive MRI solves the problem by providing 3D images of the internal structure of cuttings (Supplementary Videos S1, S2). MRI has unlimited penetration into plant tissues, uses safe radiofrequency pulses, and is more environmentally friendly, compared with X-ray tomography (Mooney *et al.*, 2012), especially for applications on meristematic and growing tissues.

Using MRI with a resolution of 30 µm, it is possible to precisely locate regions of interest such as primordia and surrounding tissue, or even changes in cambial activity and changes in the shoot’s vasculature, as early as 3 d after the excision. The MRI data can be displayed as a virtual 2D cross-section comparable to optical images (Figs 3, 4). This allows more detailed histological studies of the regions of interest (Fig. 5), as well as other spatial approaches such as FTIR spectroscopy (Fig. 6). The cumulative effect increases the power of the images. In our experiment, 3D MRI captured tissue reorganization during AR formation in the periphery of primary vascular bundles, visualized RPs in early developmental stages, and how they elongate within the stem cortex (Figs 3, 4). Both genotypes, the difficult- and easy-to-root one, bored a number of primordia, but histological and FTIR studies indicate significant differences in the cell wall characteristics of the surrounding cortical structures. This led to the conclusion that RP formation is not the limiting factor for AR formation in the MT genotype, but rather a combination of developmental stages and the inability of the ARs to efficiently penetrate and break through the cortex and epidermal cell layers of the stem.

It remains to be investigated, whether the developing ARs influence the cell wall composition of adjacent cortex cells, or if the observed probable differences in the cortex cell structure are imposed by the genetic background. Parallels are imaginable during radicle emergence through endosperm tissue, where cell wall loosening is induced by enzymatic hydrolysis, e.g. by β-1,3-glucanases (Leubner-Metzger, 2002), β-1,4-mannanases (Nonogaki *et al.*, 2000), or hydroxyl radicals (Müller *et al.*, 2009).

The ability of MRI to visualize roots in their natural environment opens great prospects for further applications (van Dusschoten *et al.*, 2016; Pflugfelder *et al.*, 2017). The introduction of multiparametric MRI methods and advanced diffusion MRI techniques for root investigation can provide important information on the dynamics of AR formation in plants *in vivo*, a kind of spatiotemporal ‘virtual histology’ (Morozov *et al.*, 2017). The great progress in plant MRI that has been achieved in the last decade (Borisjuk *et al.*, 2023) and the excellent sub-cellular resolution of modern high-field MRI (van Schadewijk *et al.*, 2020) point to new avenues for the study of root development and physiology in crop plants. Furthermore, our results demonstrate the ability to identify biochemical drivers to limit AR formation in rose using an outstanding combination of imaging techniques. This may be applicable to other root-recalcitrant species in the future, and offers the possibility to effectively improve AR formation.

## Supplementary data

The following supplementary data are available at *JXB* online.

Fig. S1. Schematic representation of methods used for the investigation of AR formation at different developmental stages of AR formation and from different genotypes.

Fig. S2. Schematic representation of the procedure for quantifying fluorescence intensities resulting from immunolabelling of different cell wall carbohydrates using ImageJ.

Fig. S3. Effect of IBA on AR formation in genotypes HF and MT for repetition 2.

Fig. S4. Monomer components showing significant correlations between percentage AR formation and relative absorbance of components in shoot bases for 15 rose genotypes.

Fig. S5. Characteristic FTIR spectra fingerprint clusters and cell wall carbohydrates and lignin detected in cross-sections of genotypes HF and MT.

Fig. S6. Characteristic FTIR spectra fingerprint clusters and protein compounds detected in cross-sections of genotypes HF and MT.

Fig. S7. Immunolocalization of hemicellulosic epitopes in cross-sections of the difficult-to-root genotype MT and the easy-to-root genotype HF.

Fig. S8. Lignin staining of shoot base cross-sections via improved Mäule staining.

Video S1. MRI of exogenous structures (left), AR formation associated structures (middle), and AR formation associated structures embedded in surrounding microshoot tissue (right) in genotype HF.

Video S2. MRI of exogenous structures (left), AR formation associated structures (middle) and AR formation associated structures embedded in surrounding microshoot tissue (right) in genotype MT.

Table S1. Rooting performance characteristics for 15 rose genotypes under different conditions *in vitro*.

Table S2. Variance analysis results for morphological AR formation and histological root primordia formation experiments.

Table S3. Phenotypic data and summary statistics results for adventitious root formation experiments for combined analysis of repetition 1 and 2.

Table S4. Statistical test results for differences in RP dimensions and cell density in RP formation experiments.

Table S5. Radial vasculature ring expansion and cell density of NMR analysed shoots.

Table S6. Relative percent difference (RPD) values for ATR-FTIR referenced components and Pearson’s correlation coefficients (R) with different AR formation traits and related *P*-values.

Table S7. Summary statistics on spectra fingerprinting data for clusters 5 to 7 for hemicellulose units.

Table S8. Summary statistics on spectra fingerprinting data for clusters 5 to 7 for cell wall carbohydrates and different lignin units.

Table S9. Summary statistics on spectra fingerprinting data for clusters 5 to 7 for proteins.

erae158_suppl_Supplementary_Tables_S1-S9

erae158_suppl_Supplementary_Figures_S1-S8

erae158_suppl_Supplementary_Video_S1

erae158_suppl_Supplementary_Video_S2

## Data Availability

All primary data to support the findings of this study are openly available in the Research Data Repository of Leibniz University Hannover at https://doi.org/10.25835/mzgm5oml (Wamhoff *et al*., 2024).

## Abbreviations:

abs: absorbance
AFA: alcohol-formalin-acetic acid
AR: adventitious root
ATR: attenuated total reflection
BSA: bovine serum albumin
EMSC: extended multiplicative signal correction
FTIR: Fourier-transform infrared
Gal: galactans
GalARha: rhamnogalacturonan
HF: ‘Herzogin Friederike’
IBA: indole-3-butyric acid
Man: mannans
MRI: magnetic resonance imaging
MT: ‘Mariatheresia’
NMR: nuclear magnetic resonance
PBS: phosphate-buffered saline
RAA: rhodamine B - acriflavin - astra blue staining
RP: root primordium
RPs: root primordia
Xyl: xylans
