# Supplementary material for: Anatomical limitations in adventitious root formation revealed by magnetic resonance imaging, infrared spectroscopy, and histology of rose genotypes with contrasting rooting phenotypes
Source: J Exp Bot. 2024 Apr 12;75(16):4784–801. doi: 10.1093/jxb/erae158 (PMC11350080; doi:10.1093/jxb/erae158)
Supplement: erae158_suppl_Supplementary_Figures_S1-S8 [file erae158_suppl_supplementary_figures_s1-s8.pdf]

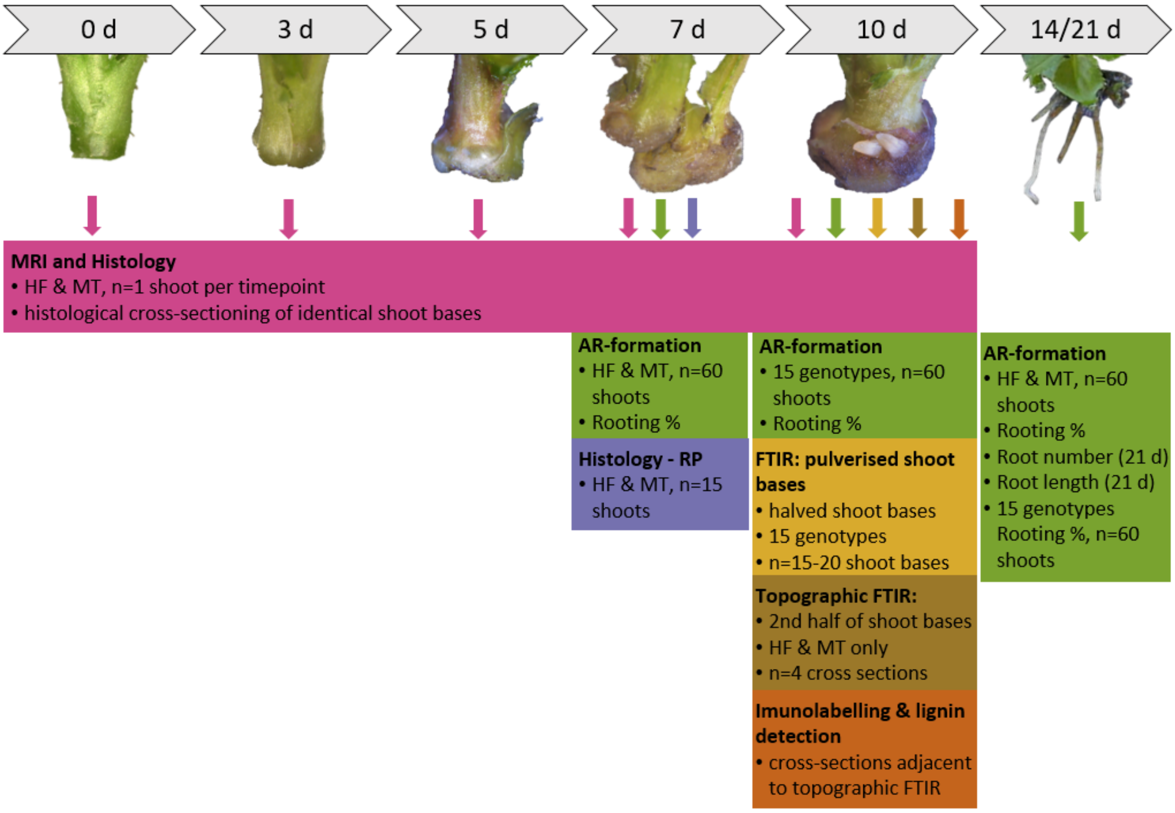

Figure S1. Schematic representation of methods used and for the investigation of AR formation on plant material of different developmental stages of AR formation and of different genotypes.

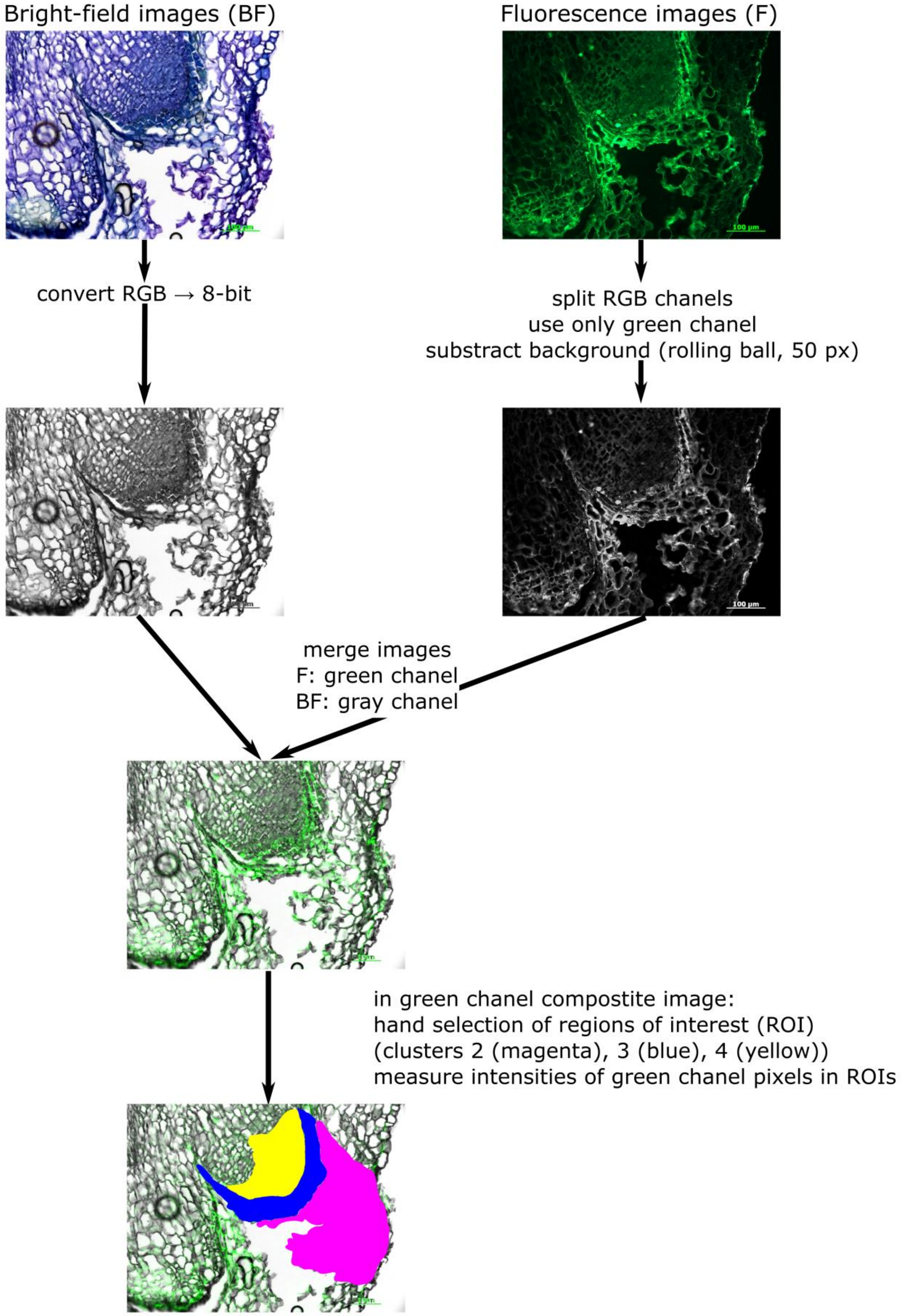

Figure S2. Schematic representation of the procedure for quantifying fluorescence intensities resulting from immunolabeling of different cell wall carbohydrates using ImageJ.

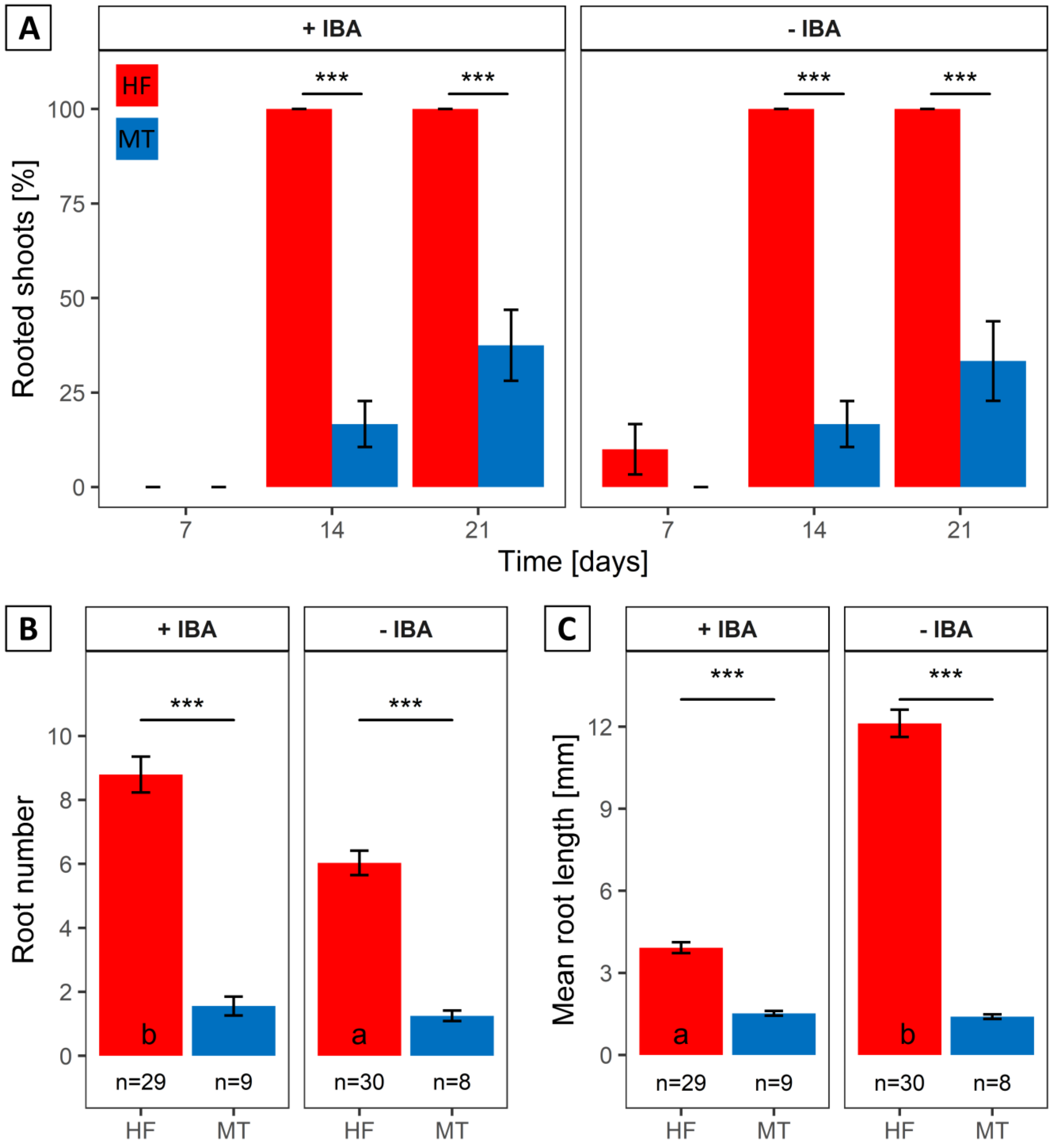

Figure S3. Effect of IBA on AR formation of cultivars HF and MT for repetition 2. Genotype performance after 3 weeks of cultivation on rooting media with (+IBA, 0.98 µM) and without IBA (–IBA). (**A**) Mean rooting percentages [%] after 7, 14, and 21 d of cultivation for five vessels with six shoots each. (**B**) Root number per rooted shoot after 21 d of cultivation, *n* displays the number of rooted shoots per genotype and IBA treatment. (**C**) Mean root length [mm] per rooted shoot after 21 d of cultivation, *n* displays the number of rooted shoots per genotype and IBA treatment. Small coloured letters indicate significant differences ($P<0.05$, Tukey's test) between the IBA variants within one genotype, while asterisks indicate significant differences between the genotypes within one IBA variant, * $p<0.05$, ** $p<0.01$, *** $p<0.001$. Tukey's test, $\alpha=0.05$.

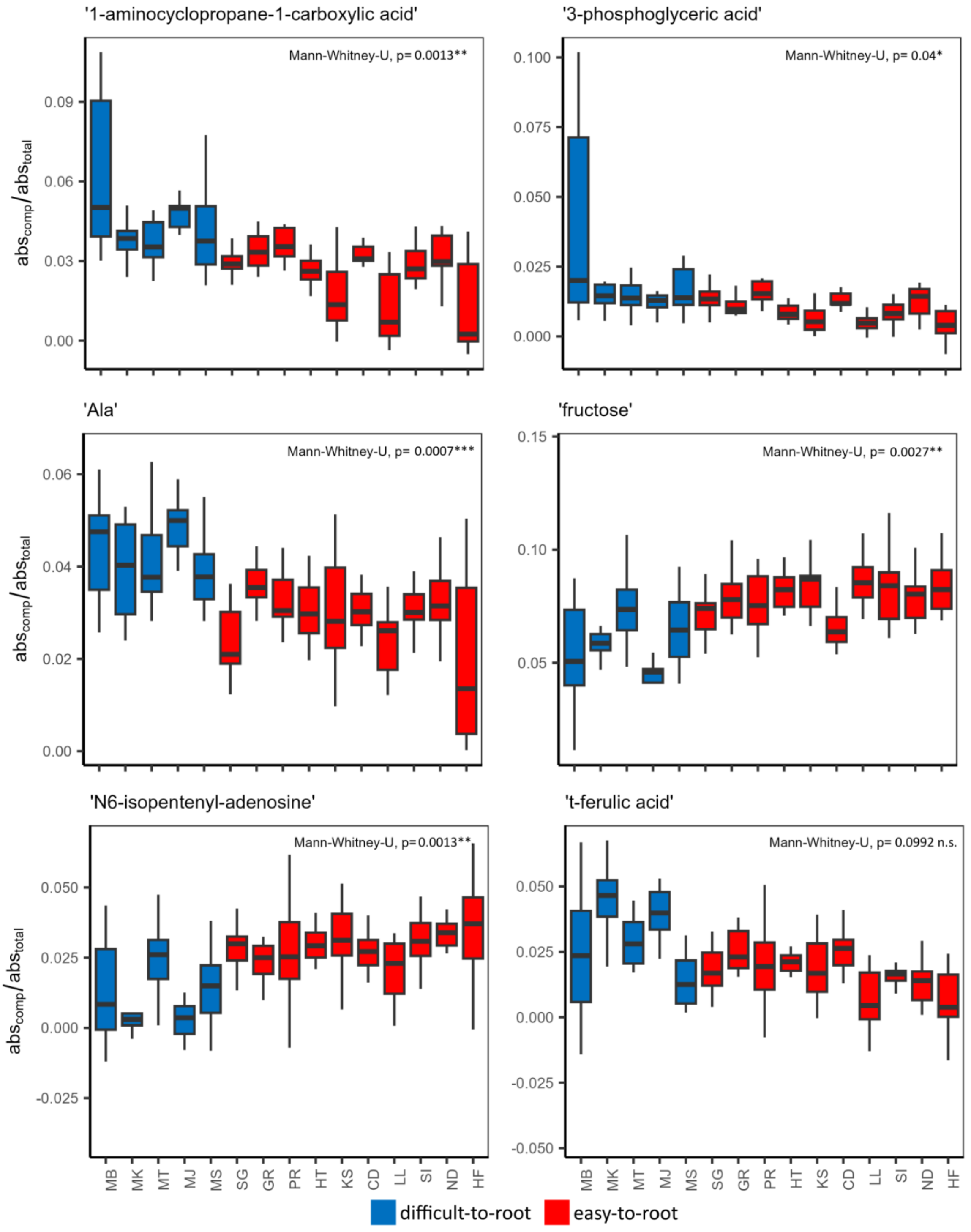

Figure S4. Monomer components showing significant correlations between AR formation percentages and relative absorbance of components' in shoot bases for 15 rose genotypes. The Y-axis shows the absorbance for the specific compound in relation to the total absorbance. Only compounds with a relative percent difference of RPD>3 (Supplementary Table S6), significant Pearson´s correlation coefficients (*P*>0.05; Supplementary Table S6) between rooting performances and absorbances, and significant Mann-Whitney-U results between relative absorbances for groups of difficult- (blue) and easy-to-root (red) genotypes are shown. Genotypes are ordered according to their increasing mean AR performance rank (Supplementary Table S1).

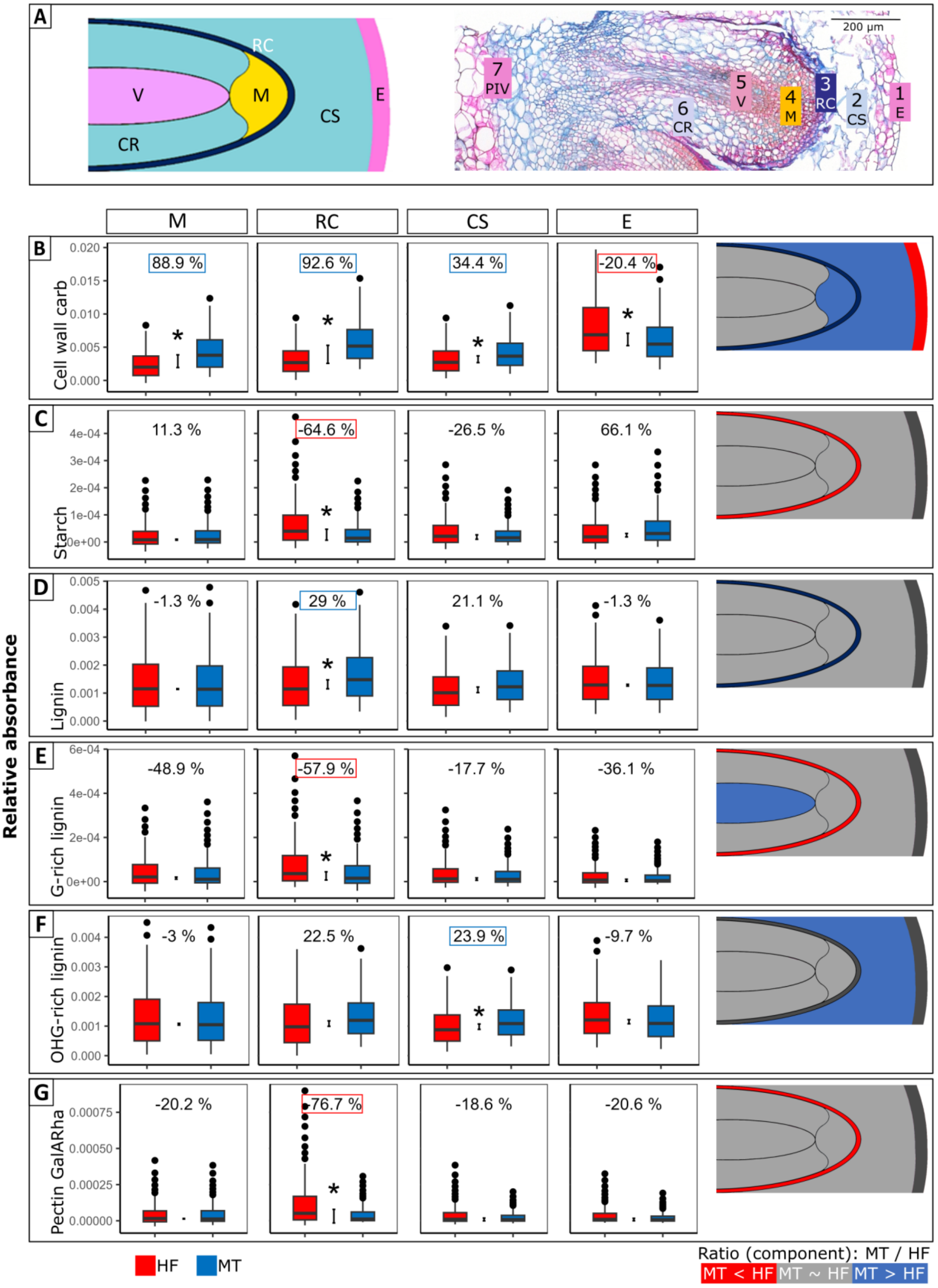

Figure S5. Characteristic FTIR spectra fingerprint clusters and cell wall carbohydrates and lignin detected in cross-sections of genotypes HF and MT. (**A**) Scheme of the characteristic seven (1-7) clusters within a rose shoot base cross-section generated by hierarchical clustering of fingerprints (left) and localisation in a cross-section of genotype MT after 10 days on +IBA (0.98 µM) medium; representing functional tissues of 1 E- epidermis, 2 CS- cortex shoot, 3 RC- root cap, 4 M- meristematic

region, 5 V- vasculature, 6 CR- cortex root, 7 PIV- pith and vascular ring. (**B-G**) Relative absorbance for cell wall carbohydrates, starch, lignin, guaiacyl rich lignin (G-rich lignin), 5-hydroxyguaiacyl rich lignin (OHG-rich lignin), and pectin rhamnogalacturonan (GalARha) for genotypes HF and MT for the clusters 1 to 4, representing M, RC, CS and E from left to right. Boxplot graphs show median values as thick black horizontal lines, interquartile range (IQR) as coloured box, and whiskers representing 1.5 × IQR, error bars show the quantile absolute difference (QAD), presence of * indicates that the difference between the two distributions was significant (Mann-Whitney *p*-value<0.001) and the divergence effect size was >0.8 or R^2 of the divergence effect size was >0.5. Colouring in cluster in schemes indicates significant differences between the two tested genotypes within the single clusters. Grey colour indicates no difference, red colour indicates lower and blue colour higher values for MT compared to HF. The values in per cent indicate the ratio of the difference between the values of MT and HF (MT-HF) in relation to the value for HF as 100%.

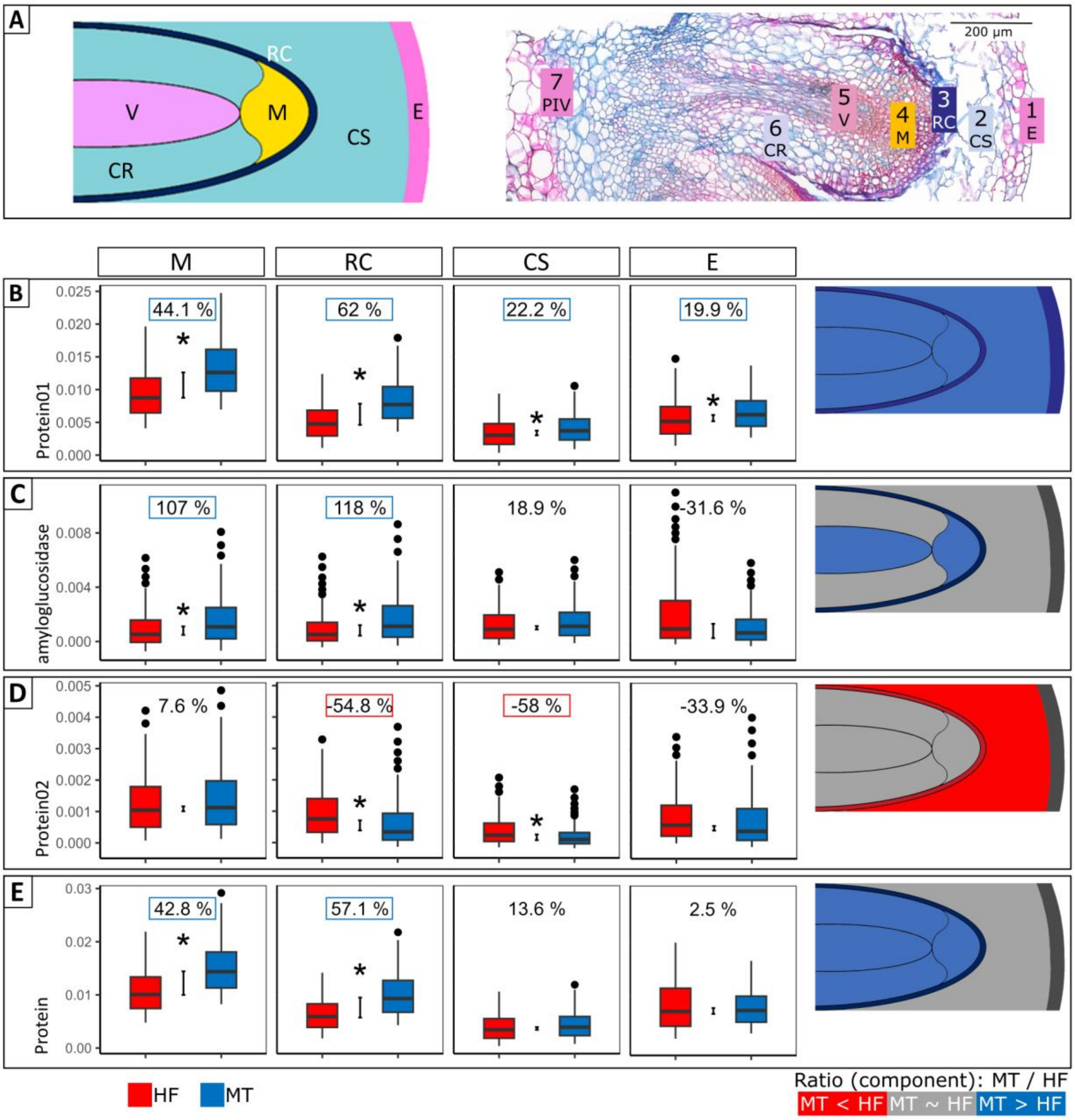

Figure S6. Characteristic FTIR spectra fingerprint clusters and protein compounds detected in cross-sections of genotypes HF and MT. (**A**) Scheme of the characteristic seven (1-7) clusters within a rose shoot base cross-section generated by hierarchical clustering of fingerprints (left) and localisation in a

cross-section of genotype MT after 10 days on +IBA (0.98 µM) medium; representing functional tissues of 1 E- epidermis, 2 CS- cortex shoot, 3 RC- root cap, 4 M- meristematic region, 5 V- vasculature, 6 CR- cortex root, 7 PIV- pith and vascular ring. (**B-E**) Relative absorbance for proteins of type 1, amyloglucosidase, proteins of type 2, and all proteins in general for genotypes HF and MT for the clusters 1 to 4, representing M, RC, CS and E from left to right. Boxplot graphs show median values as thick black horizontal lines, interquartile range (IQR) as coloured box, and whiskers representing 1.5 × IQR, error bars show the quantile absolute difference (QAD), presence of * indicates that the difference between the two distributions was significant (Mann-Whitney *p*-value<0.001) and the divergence effect size was >0.8 or R^2 of the divergence effect size was >0.5. Colouring in cluster in schemes indicates significant differences between the two tested genotypes within the single clusters. Grey colour indicates no difference, red colour indicates lower and blue colour higher values for MT compared to HF. The values in per cent indicate the ratio of the difference between the values of MT and HF (MT-HF) in relation to the value for HF as 100%.

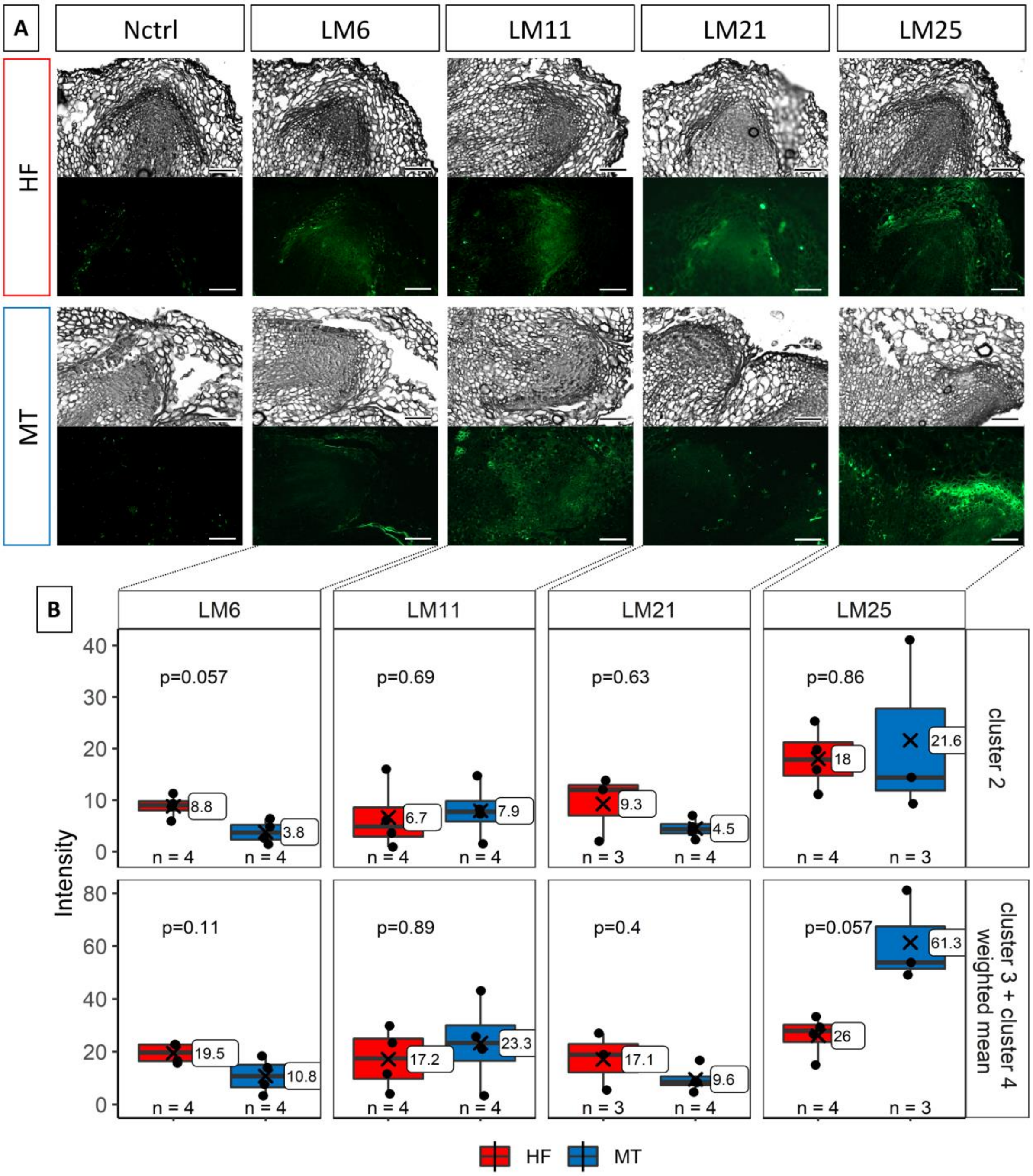

Figure S7. Immunolocalisation of hemicellulosic epitopes in cross-sections of the difficult-to-root genotype MT and the easy-to-root genotype HF. (**A**) Cross-section through the shoot base with an AR primordium (lower panel) and under fluorescent light after immunostaining (upper panel) with mAbs LM6 (anti-arabinan), LM11 (anti-xylan/arabinoxylan), LM21 (anti-mannan) and LM25 (anti-xyloglucan) or without immunostaining (nctrl) for genotypes HF and MT. Bars: 100 µm. (**B**) Quantified fluorescence intensities in the pre-defined clusters 2-4 for genotypes HF and MT and distinct mAb-treatments. Mean fluorescence intensities for cluster 2 and combined for clusters 3 and 4 are displayed. The number of analysed root primordia per genotype is indicated by *n*, X and the numbers show the mean. *P*-values are given for results of Wilcoxon rank sum test. The images for LM25 in (**A**) are also shown in Fig. 7. Abbreviations: cs - cortex shoot; cr - cortex root; e – epidermis; m – meristem; rc – root cap; v - vascular tissue.

A

Fluoresc. pattern	AR developmental stage HF: 1		2		3		Σ_{HF}		MT: 1		2		3		Σ_{MT}	
A	0	0%	0	0%	0	0%	0	0%	1	8%	1	8%	0	0%	2	15%
B	3	30%	0	0%	1	10%	4	40%	2	15%	2	15%	2	15%	6	46%
C	0	0%	2	20%	0	0%	2	20%	2	15%	2	15%	0	0%	4	31%
D	4	40%	0	0%	0	0%	4	40%	1	8%	0	0%	0	0%	1	8%
Σ	7	70%	2	20%	1	10%	10	100%	6	46%	5	38%	2	15%	13	100%

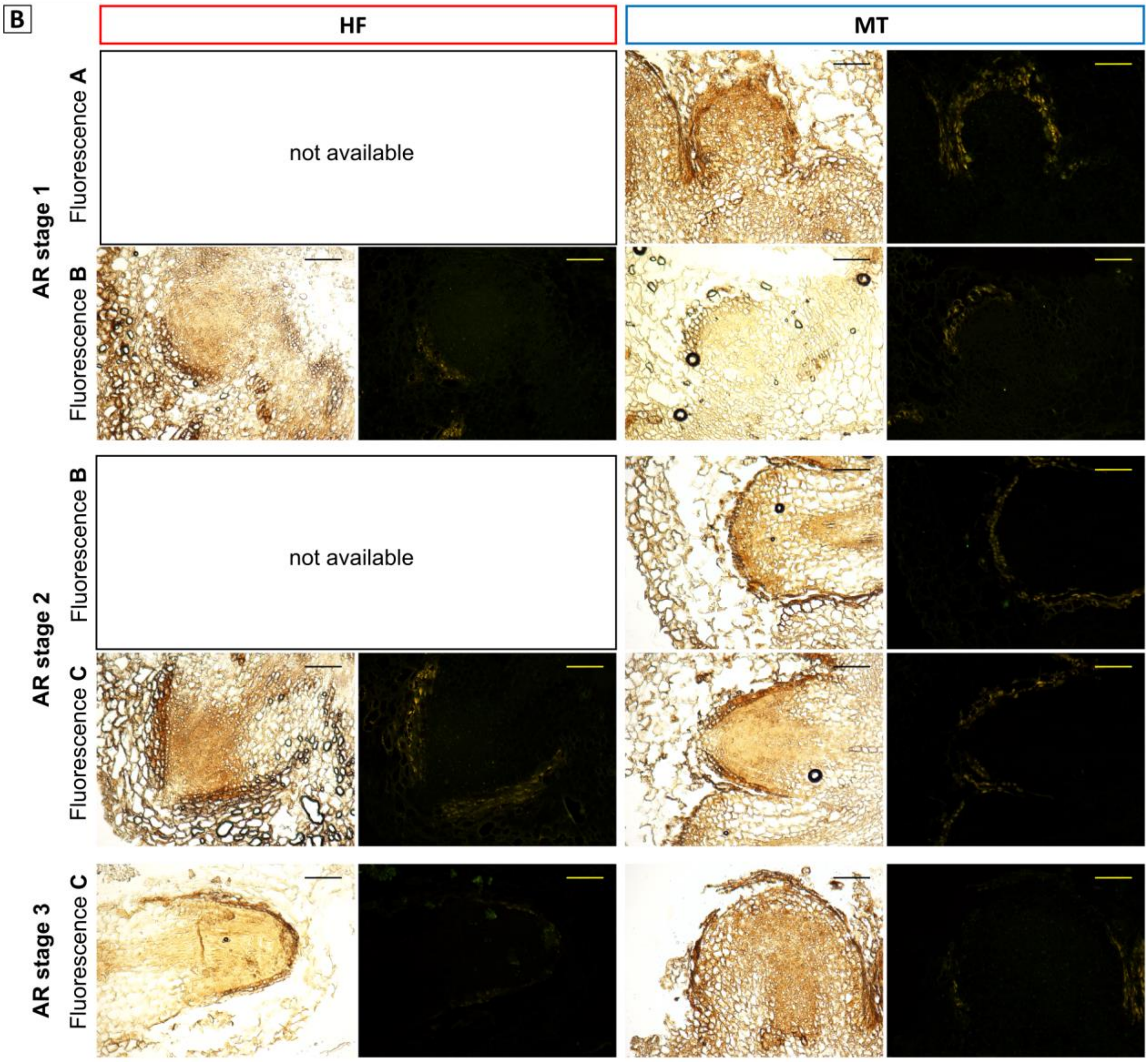

Figure S8. **Lignin staining of shoot base cross sections via improved Mäule staining.** (**A**) Categorised counting data dividing the root primordia into three different developmental stages (columns; 1 - dome-shaped root primordium, 2 - root primordium with vascular system, 3 - outgrowing fully developed root) and the observed fluorescence in the root cap (cluster 3) into four distinct fluorescence patterns (rows; A - full root cap strong fluorescent, B - whole root cap light fluorescent, C - only parts of root cap fluorescent, D - no fluorescence). Numbers of observed root primordia per AR developmental stage and fluorescence pattern were counted and percentages in relation to total number of analysed root primordia were calculated for genotypes HF and MT, respectively. (**B**) Cross sections of genotypes HF and MT stained with improved Mäule stain showing different AR developmental stages by light microscopy images (left) and corresponding fluorescence images (right) showing characteristic patterns in cluster of root cap.
